# Supplementary material for: Data-driven method to infer the seizure propagation patterns in an epileptic brain from intracranial electroencephalography
Source: PLoS Comput Biol. 2021 Feb 17;17(2):e1008689. doi: 10.1371/journal.pcbi.1008689 (PMC7920393; doi:10.1371/journal.pcbi.1008689)
Supplement: S1 Text — (PDF) [file pcbi.1008689.s001.pdf]

Supplementary information: Data-driven method to infer the seizure propagation patterns in an epileptic brain from intracranial electroencephalography

Viktor Sip[a], Meysam Hashemi[a], Anirudh N. Vattikonda[a], Marmaduke M. Woodman[a], Huifang Wang[a], Julia Scholly[b,c], Samuel Medina Villalon[a,c], Maxime Guye[b,d], Fabrice Bartolomei[a,c], and Viktor K. Jirsa[a,*]

[a] *Aix Marseille Univ, INSERM, INS, Inst Neurosci Syst, Marseille, France*
[b] *Assistance Publique - Hôpitaux de Marseille, Hôpital de la Timone, CEMEREM, Pôle d'Imagerie Médicale, CHU, Marseille, France*
[c] *Assistance Publique - Hôpitaux de Marseille, Hôpital de la Timone, Service de Neurophysiologie Clinique, CHU, Marseille, France*
[d] *Aix Marseille Univ, CNRS, CRMBM, Marseille, France*
[*] *viktor.jirsa@univ-amu.fr*

Note on parameter identifiability

Investigation of the properties of the dynamical model can shed light on the identifiability of the parameters of the single-seizure statistical model (Box 2 in the main text) in the limit case of no observation noise $\sigma_t = 0$. Recall that the dynamical model reads

$$\dot{z}_i = f_{\boldsymbol{q}} \left(c_i, \; \sum_{j=1}^{n} w_{ij} H(z_j - 1) \right), \quad z_i(t_0) = 0 \qquad \text{for } i = 1, \ldots, n \tag{1}$$

with $t_0 = 0$, and that the onset time of a region i is defined as $t_i = \min\{t \mid z_i(t) \geq 1\}$. Recall also that we use the shorthand $P_{W,\boldsymbol{q}}(\boldsymbol{c}) = \boldsymbol{t}$ for the mapping between the excitabilities $\boldsymbol{c} = (c_1, \ldots, c_n)$ and onset times $\boldsymbol{t} = (t_1, \ldots, t_n)$ defined by the dynamical model (1) with the connectome matrix W and parameterization $\boldsymbol{q}$.

Theorem 1. *Let $f_{\boldsymbol{q}}(c, y) : \mathbb{R} \times [0, 1] \to \mathbb{R}^+$ be a continuous function, strictly increasing in c and onto $\mathbb{R}^+$ for any y. Then $P_{W,\boldsymbol{q}} : \mathbb{R}^n \to (\mathbb{R}^+)^n$ is one-to-one and onto.*

Proof. Since $z_i(t_0) = 0$ and $z_i(t)$ is increasing in t due to the positive RHS of (1), it follows from the definition that $t_i \in \mathbb{R}^+$ for all $i = 1, \ldots, n$. One thus has to show that for every $\boldsymbol{t} \in (\mathbb{R}^+)^n$ there exists one and only one $\boldsymbol{c} \in \mathbb{R}^n$ that satisfies $P_{W,\boldsymbol{q}}(\boldsymbol{c}) = \boldsymbol{t}$.

Assume that there is a $\boldsymbol{t} = (t_1, t_2, \ldots, t_n) \in (\mathbb{R}^+)^n$, and assume, without loss of generality, that it is ordered so that $0 < t_1 \leq t_2 \leq \ldots \leq t_n$. If not, one can simply reorder the indices of the regions. Since the RHS in (1) is constant for $t \in [t_{j-1}; t_j)$ for any $j = 1, \ldots, n$, one can write

$$\begin{aligned} z_i(t_j) &= z_i(t_{j-1}) + (t_j - t_{j-1}) f_{\boldsymbol{q}} \left(c_i, \sum_{k=1}^{n} w_{ik} H(z_k(t_{j-1}) - 1) \right) \\ &= z_i(t_{j-1}) + (t_j - t_{j-1}) f_{\boldsymbol{q}} \left(c_i, \sum_{k=1}^{j-1} w_{ik} \right) \\ &= z_i(t_0) + \sum_{l=1}^{j} (t_l - t_{l-1}) f_{\boldsymbol{q}} \left(c_i, \sum_{k=1}^{j-1} w_{ik} \right) \\ &= \sum_{l=1}^{j} (t_l - t_{l-1}) f_{\boldsymbol{q}} \left(c_i, \sum_{k=1}^{l-1} w_{ik} \right). \end{aligned} \tag{2}$$

The second equality follows from the definition of t_k implying that $z_k(t) < 1$ for $t < t_k$ and from the ordering of $\boldsymbol{t}$. By setting $i = j$ we obtain

$$1 = z_i(t_i) = \underbrace{\sum_{l=1}^{i} (t_l - t_{l-1}) f_{\boldsymbol{q}} \left(c_i, \sum_{k=1}^{l-1} w_{ik} \right)}_{g_i(c_i)} \qquad \text{for } i = 1, \ldots, n. \tag{3}$$

Since $(t_l - t_{l-1})$ are non-negative (with at least $t_1 - t_0$ being positive) and $f_{\boldsymbol{q}}$ is strictly increasing in c and onto $\mathbb{R}^+$, it follows that g_i is also strictly increasing and onto $\mathbb{R}^+$, therefore (3) has a unique solution for all $i = 1, \ldots, n$. $\square$

This theorem helps to make a clear link between the observations and the model parameters. In an observation of a spreading seizure in a network any region i can be either

- observed seizing, in which case it has a known onset time t_i;
- or observed non-seizing, which we represent as having an onset time $t_i \geq t_{\lim}$;
- or hidden.

If we reorder the regions in this order, any particular observation of a seizure can be represented as a set T in the space of observations $(\mathbb{R}^+)^n$,

$$T = \underbrace{\{t_1\} \times \ldots \times \{t_{n_{\mathrm{sz}}}\}}_{\text{Seizing regions, } n_{sz}} \times \underbrace{[t_{\lim}, \infty) \times \ldots \times [t_{\lim}, \infty)}_{\text{Non-seizing regions, } n_{ns}} \times \underbrace{\mathbb{R}^+ \times \ldots \times \mathbb{R}^+}_{\text{Hidden regions, } n_{hid}} \tag{4}$$

This is an $(n_{ns} + n_{hid})$-dimensional manifold, and if $n_{ns} > 0$, then it has an $(n_{ns} + n_{hid} - 1)$-dimensional boundary representing the limit cases of one non-seizing region having the onset time $t_{\lim}$. And since $P_{W,\boldsymbol{q}}$ is one-to-one and onto, $P^{-1}_{W,\boldsymbol{q}}$ exists, and $P^{-1}_{W,\boldsymbol{q}}(T)$ is a manifold of the same dimensionality in the space of excitability parameters $\mathbb{R}^n$. This parameter manifold represents all possible solutions to the inverse problem for a particular observation T. The probability of the solutions on the manifold is then determined by the prior distribution on the parameters. Fig 1 illustrates this in a three-node network.

Validation on synthetic data

To instill confidence in the inference process and to establish what can be expected from the inference from the incomplete seizure observations, we first performed extensive validation of the method on synthetic data, i.e. on data generated by the same dynamical model which was then used for the inference. For three different functions $f_{\boldsymbol{q}}$ (with no coupling, weak coupling, or strong coupling; Fig 2) we generated synthetic seizures by randomly sampling the excitabilities from standard normal distribution and calculating the region onset times using the dynamical model. If there were no seizing regions in the simulated seizure (i.e. no regions with onset time $t_i < t_{\lim}$ with $t_{\lim} = 90\,\mathrm{s}$), a new seizure with new excitability parameters was generated. The parameters $\boldsymbol{q}$ of the excitation functions were selected by hand, loosely guided by the principle of preserving some key statistics of generated seizures (Fig 2B). The exception was the strong coupling function, which was in fact the result of the inference performed on real data; the results for strong coupling thus provide an estimate of what can be expected in real data. We then ran the multi-seizure and single-seizure inference on these synthetic data, assuming that only portion of the regions (21, 54, or 108 out of 162) were observed, and we evaluated how well were the original parameters recovered. For every seizure, first one observed region was selected from those seizing, and then the other observed regions were selected randomly from all regions; this was to assure that there was at least one region seizing among those observed.

To test the recovery of the hyperparameters $\boldsymbol{q}$, we ran the multi-seizure model on batches of twelve synthetic seizures. Four independent MCMC chains were run for each batch; out of total of 36 chains one chain was stuck and was excluded from further analysis. The convergence was evaluated using the split-chain reduction factors $\hat{R}$ and number of effective samples N_{eff} [1]. All but one scenarios converged satisfactory with $\hat{R}$ below 1.1 and with N_{eff} above 30 for

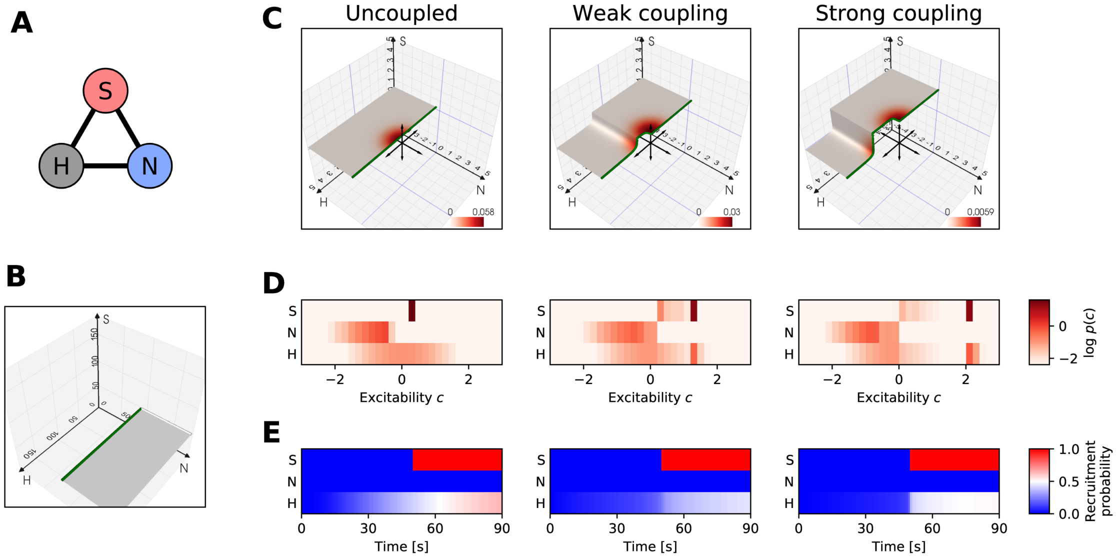

Fig 1. Observation and parameter manifolds in a three-node network. (A) Three node network with one hidden, one observed non-seizing, and one observed seizing region (with $t_S = 50\,\mathrm{s}$), marked with H, N, and S respectively. The network is fully and symmetrically connected with connection strengths $w_{ij} = 0.1$. (B) The observation manifold in the onset time space $(\mathbb{R}^+)^3$. The green line marks the limit case of $t_N = t_{\mathrm{lim}}$. (C) The parameter manifolds in the parameter space $\mathbb{R}^3$ for three excitation functions $\boldsymbol{f_q}$ representing no, weak, and strong coupling (see Fig 2). In general, the dimensionality of the manifolds is determined by the number of seizing, non-seizing, and hidden regions in the network, while the shape of the manifold is determined by the connectivity W, excitation function $\boldsymbol{f_q}$, and the onset times of seizing regions. The coloring represents the prior distribution of the excitability parameters, which is a standard normal distribution $N(\mathbf{0}, \boldsymbol{I})$. (D) Marginal posterior probabilities of the excitability parameters of the three nodes, obtained by integrating the prior over the manifold. (E) Recruitment probabilities of the three regions. With no observation noise, the only differences are in the hidden region H, and the observations of the other two regions are reproduced exactly. This figure illustrates how the stronger coupling leads to more complicated posterior geometry (C), possibly resulting in a multimodality in the distribution of the excitability parameters (D). In this case, in addition to the dominant mode representing the highly excitable observed region S, the strong coupling introduces also a secondary mode representing the highly excitable hidden region H and less excitable observed region S. In other words, the coupling allows for a discovery of a hidden epileptogenic zone.

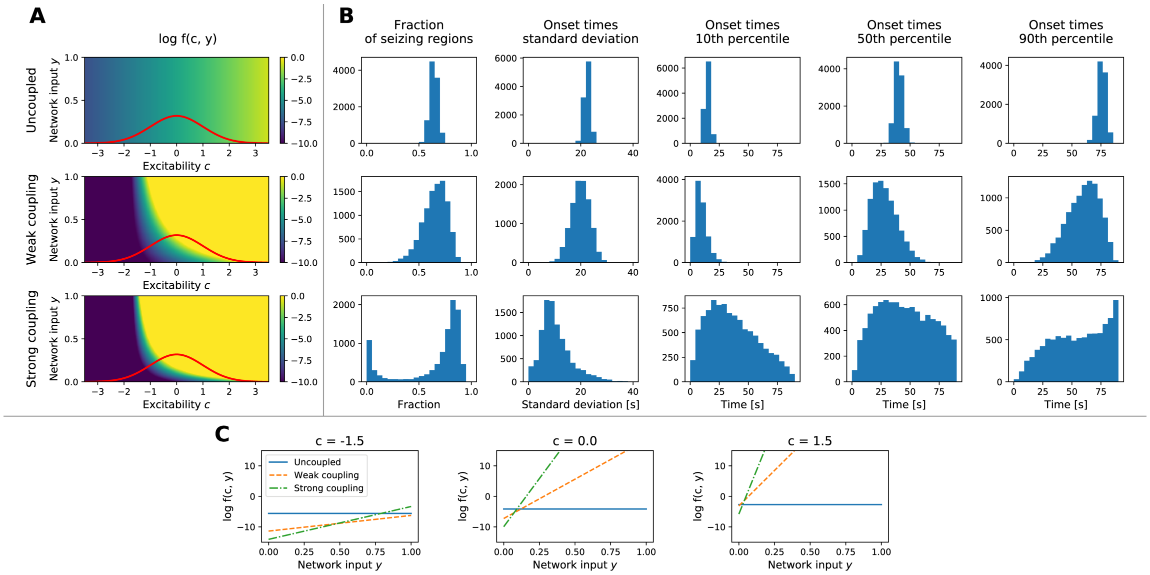

Fig 2. Three excitation functions used for the synthetic data validation. (A) Plots of the functions $f_{\boldsymbol{q}}(c, y)$ named "Uncoupled" with $\boldsymbol{q} = (-5.12, -5.12, 1.95, 1.95)$, "Weak coupling" with $\boldsymbol{q} = (-10.0, 2.0, 5.5, 33.0)$, and "Strong coupling" with $\boldsymbol{q} = (-12.70, 15.48, 5.53, 75.21)$. The colormap is clipped to show the range of values relevant for the seizure propagation on timescales of seconds to tens of seconds. The red line shows the prior distribution on the region excitabilities c, i.e. the standard normal function. (B) Statistical quantification of the generated seizures. For each function (in rows) 10000 seizures were generated by taking a random patient connectome and randomly sampling excitabilities using the standard normal distribution. Columns show the histograms of properties of the generated seizures: fraction of seizing regions, standard deviation of the onset times of seizing regions, and percentiles of the onset times of seizing regions. Vertical axis always represents the count of seizures. (C) Dependency of the excitation functions on the network input for different values of excitability. The stronger or weaker relation motivates our notation of Uncoupled, Weak and Strong coupling for the different functions.

all hyperparameters. The exception was the scenario with the strong coupling function with 108 observed regions ($\hat{R} \leq 1.41$, $N_{eff} \geq 5.79$); these results are nevertheless shown as well. We adopt the evaluation framework of Betancourt [2] and present the values of posterior z-score and posterior shrinkage. For a parameter θ_i, the posterior z-score quantifies how far is the posterior from the ground truth,

$$z_i = \left| \frac{\bar{\theta}_i - \theta_i^*}{\sigma_{i,post}} \right|, \tag{5}$$

where $\bar{\theta}_i$ is the mean of the posterior distribution, θ_i^* is the ground truth, and $\sigma_{i,post}$ is the standard deviation of the posterior distribution. The posterior shrinkage quantifies how much is the uncertainty in the prior distribution reduced,

$$s_i = 1 - \frac{\sigma_{i,post}^2}{\sigma_{i,prior}^2}, \tag{6}$$

where $\sigma_{i,prior}$ is the standard deviation of the prior distribution. Fig 3A-C shows the results. Most data points are located in the lower right corner, indicating good recovery of the parameters. Some overfitting however can be seen for the uncoupled model; considering that the shrinkage values are very close to one, the distance is still small in the absolute numbers. This overfitting may be caused by the fact that the data were generated without any observation noise assumed in the statistical model, leading to more confident estimations.

To test the recovery of the low-level excitability parameters we ran the single-seizure model on synthetic seizures, assuming the knowledge of the hyperparameters $\boldsymbol{q}$ used for their generation. We have again used the same three sets of the hyperparameters and for each of them we generated 96 seizures, again sampling the excitability parameters randomly from a standard normal distribution, and using the connectomes from the patient cohort. One chain out of total of 1728 was stuck and was excluded. The vast majority of the inference runs converged well, with 99.84% regional excitability parameters with $\hat{R}$ below 1.1 and N_{eff} above 30. We evaluated the relation between the ground truth and the mean of the posterior distribution, separately for the observed (Fig 3D) and hidden nodes (Fig 3E). For the observed nodes, best values are obtained in the uncoupled network, and the goodness of fit is reduced with stronger coupling; the effect of added observed nodes is minimal. For the hidden nodes the relation between the posterior mean and ground truth is in general much weaker. When the network is entirely uncoupled, nothing can be inferred about the hidden nodes. With stronger coupling some information can be extracted, with positive effect of added observed nodes. Interestingly, too strong coupling decreases the goodness of fit. In such scenarios the network acts homogeneously, and it is difficult to infer which node is the driver and which is the follower. Fig 3F and 3G shows how well can be the seizing/non-seizing state and the onset times of hidden nodes predicted. Here the relation is clear: stronger coupling leads to better predictions, with small benefits of adding observed nodes.

Discovery of epileptogenic zones in synthetic seizures

Before applying the method to discover the epileptogenic zone in the patient recordings, we tested how well can the epileptogenic zones be discovered in the synthetic data. These were generated by the trained model using patients' structural connectivities and electrode implantations. In the model, we equated the epileptogenic zone with the region with high excitability $c > c_h$ for the

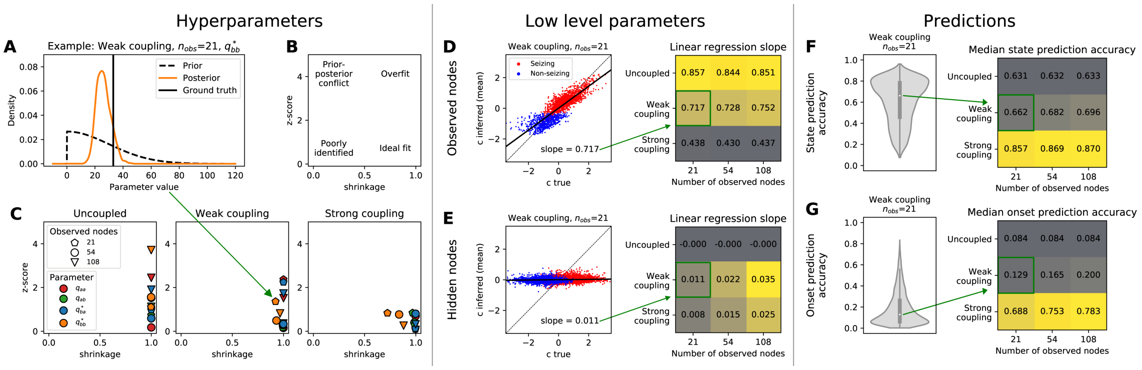

Fig 3. Validation on synthetic data. (A) Example of a hyperparameter inference. The posterior is more concentrated around the ground truth compared to the prior. (B) Interpretation of the shrinkage - z-score plot. Lower right corner constitutes the ideal fit. (C) Shrinkage - z-score plots for the three excitation functions, three numbers of observed nodes out of total of 162, and all four hyperparameters. (D,E) Goodness of fit for the low-level parameters. Left panels show the relation between the ground truth and the posterior mean of the excitability in one simulated scenario with weak coupling and 21 observed nodes. Each dot represent one brain region, colored based on whether it seizes or not. The dashed line represents a perfect fit and the solid line shows the linear regression fit. Right panels show the linear regression slope for all scenarios. Note that the color code is different between D and E. (F) Distribution of state prediction accuracy (i.e. of the probabilities that a seizing/non-seizing state of a hidden region is correctly inferred) for one scenario (left), and its median for all scenarios (right). (G) Distribution of onset prediction accuracy (i.e. of the probabilities that a onset time for a hidden seizing region is correctly inferred) for one scenario (left), and its median for all scenarios (right). The violin plots in F and G show a kernel density estimate of the probability density of a given variable across all regions and across all seizures for the given scenario. The inner boxplots show the median (white dot), interquartile range (IQR, gray bar) and adjacent values (upper/lower quartile +/- 1.5 IQR, gray line).

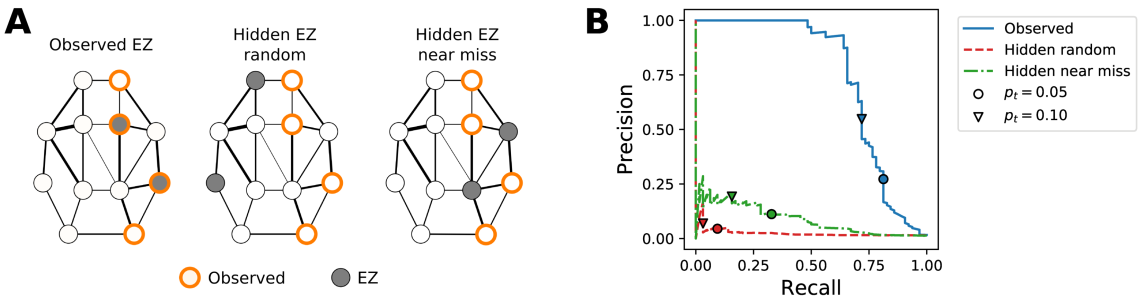

Fig 4. Discovery of the observed and hidden epileptogenic zones (EZ) in synthetic seizures. (A) Three scenarios of EZ location. In all three scenarios, two EZs (with the excitabilities drawn from the standard normal distribution bottom-truncated at $c = c_h$) are placed in the network, and the rest of the nodes has excitabilities drawn from the standard normal distribution top-truncated at $c = c_h$. In the observed EZ scenario, the EZs are placed randomly among the observed nodes. In the random hidden EZ scenario, the EZs are placed randomly among the non-observed regions. In the near miss hidden EZ scenario, the EZs are placed among the non-observed nodes that are well-connected to at least three observed nodes, where well-connected means having the outgoing connection stronger that 97-percentile of all connections weights. Thirty-two synthetic seizures were generated and fitted for each scenario. (B) Precision-recall curves obtained by using the inferred probability $p(c > c_h)$ that a region is highly excitable as a predictor and varying a threshold p_t for this predictor from zero to one. Two specific thresholds p_t are marked on each curve.

threshold $c_h = 2$. In order to establish how well can be the epileptogenic zone discovered based on its location relative to the implanted sensors, we analyzed three scenarios: observed epileptogenic zone, hidden epileptogenic zone with random location among the non-observed regions, and a *near miss* scenario, that is a hidden epileptogenic zone that is well connected to at least three observed regions (Fig 4A). We analyzed how well were the epileptogenic zones discovered with the precision-recall curve using the inferred probability of high excitability (Fig 4B). The observed epileptogenic zones were well identified, while the identification of the hidden epileptogenic zone was unsurprisingly worse. However, the location matters considerably. In the random scenario, the precision and recall for the threshold $p_t = 0.05$ is 4% and 9% respectively. In comparison, the precision and recall in the near miss scenario is at 11% and 33%, meaning that a third of the hidden epileptogenic zones were discovered with above one in ten chance of being correct. While such numbers are far from being sufficient to serve as a sole basis for any surgical decision, it might be enough to point to previously unexpected cause for which supporting evidence may (or may not) be found by other methods.

Subject-level analysis of the prediction accuracy

Here our goal is to compare the differences in leave-one-out prediction accuracies, obtained from the empirical data, among the subjects and seizures in order to see if the method performs considerably better or worse in some cases, and if so, to identify these cases. Analysis of the results on the subject level is however complicated by two main obstacles. First, as established, the values of state and

Box 1. Multi-level model for accuracy analysis

Input data: Number of subjects n^{subjects}, numbers of seizures of each subject n_i^{seizures}, numbers of analyzed regions in each seizure n_{ij}^{regions}, fractions of seizing nodes in a seizure f_{ij}, node strengths n_{ijk}, and the variable of interest y_{ijk}. Everywhere i indexes the subjects, j the seizures of a subject, and k the analyzed brain regions.
Parameters: Slopes a, b (shared globally), intercepts on a seizure level c_{ij}, on a subject level $\bar{c}_i$, and global $\bar{\bar{c}}$, auxiliary parameters $\bar{\eta}_i$ and η_{ij}, and standard deviations on a region, seizure, and subject level σ^{regions}, σ^{seizures}, σ^{subjects} (shared globally).
Model:

$$
\begin{aligned}
& a \sim \text{Normal}(0,1) \\
& b \sim \text{Normal}(0,1) \\
& \bar{\bar{c}} \sim \text{Normal}(0,1) \\
& \sigma^{\text{regions}} \sim \text{HalfNormal}(0,1) \\
& \sigma^{\text{seizures}} \sim \text{HalfNormal}(0,1) \\
& \sigma^{\text{subjects}} \sim \text{HalfNormal}(0,1) \\
\\
& \text{For } i = 1, \dots, n^{\text{subjects}}: \\
& \quad \bar{\eta}_i \sim \text{Normal}(0,1) \\
& \quad \bar{c}_i = \bar{\bar{c}} + \sigma^{\text{subjects}} \bar{\eta}_i \\
& \quad \text{For } j = 1, \dots, n_i^{\text{seizures}}: \\
& \quad\quad \eta_{ij} \sim \text{Normal}(0,1) \\
& \quad\quad c_{ij} = \bar{c}_i + \sigma^{\text{seizures}} \eta_{ij} \\
& \quad\quad \text{For } k = 1, \dots, n_{ij}^{\text{regions}}: \\
& \quad\quad\quad y_{ijk} \sim \text{Normal}(f_{ij} a + n_{ijk} b + c_{ij}, \sigma^{\text{regions}})
\end{aligned}
$$

onset prediction accuracies depend on the seizing/non-seizing state of a brain region, its connection strength, and fraction of regions that are seizing in a given seizure (Fig 6 in the main text). These factors differ between subjects and seizures, and so the values are not directly comparable and have to be corrected to account for this known dependency. Second, the number of observed regions (for which the prediction accuracies are calculated) differ between seizures and subjects, especially when analyzing separately the seizing and non-seizing regions. When this sampling variability is not accounted for, the results might give a wrong impression of variance between seizures and subjects.

To overcome these issues, we have analyzed the results using a multi-level model based on linear regression (Box 1). At the core of the model, the variable of interest is predicted via linear dependency on the fraction of seizing nodes in a seizure and node strength, thus accounting for this known relation (or rather its linear part). The slopes are shared between all seizures and subjects, while the intercepts are only partially shared between seizures and subjects through the multi-level hierarchy. This partial sharing allows to use the information from other seizures and subjects if the sample size is low, while allowing the intercepts to vary if the evidence is strong. The results for

the seizing and non-seizing regions are analyzed independently with no parameter sharing between them. Note the use of a non-centered parameterization in defining $\bar{c}_i$ and c_{ij} through the auxiliary parameters $\bar{\eta}_i$ and η_{ij}, instead of a centered parameterization (i.e. $\bar{c}_i \sim \text{Normal}(\bar{\bar{c}}, \sigma^{\text{subjects}})$ and similarly for c_{ij}). That is done to improve the efficiency of the sampling process. The priors were set as weakly informative Normal$(0, 1)$ (or HalfNormal$(0, 1)$ for positive parameters) considering that all data are on the order of one.

The model was implemented and fitted using Stan [3] utilizing Hamiltonian Monte Carlo method. Four chains were used for sampling each analyzed variable, each chain with 500 warmup and 500 sampling iterations. Standard diagnostics did not indicate any sampling issues (no divergent iterations, split chain reduction factor $\hat{R} < 1.03$ and number of effective samples $N_{eff} > 200$ for all parameters in all runs). That is in part due to the non-centered parameterization, the centered version of the same model converged considerably worse.

The results of the analysis for state and onset prediction accuracies obtained by the inference and their difference to the unweighted and weighted estimates are shown on Figs 13 and 14 respectively. As visible, individual variations from the population mean exist both on subject and seizure level; these cannot be explained by the linear dependency on node strength and fraction of seizing nodes accounted for in the model. Detailed analysis of the causes of these variations remains out of scope for this study, however, these charts can guide the future investigation of the model strengths and weaknesses on the subject and seizure level.

References

1. Gelman A, Carlin JB, Stern HS, Dunson DB, Vehtari A. Bayesian Data Analysis. Taylor & Francis Ltd; 2013.

2. Betancourt M. Calibrating Model-Based Inferences and Decisions. arXiv:180308393 [stat]. 2018;.

3. Carpenter B, Gelman A, Hoffman MD, Lee D, Goodrich B, Betancourt M, et al. Stan: A Probabilistic Programming Language. Journal of Statistical Software. 2017;76(1). doi:10.18637/jss.v076.i01.

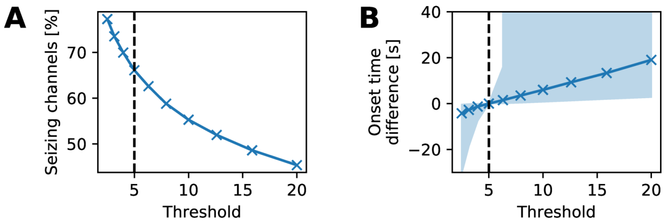

Fig 5. Influence of the threshold δ on the detection of seizing channels and their onset times. Threshold δ corresponds to δ-fold increase of the signal power compared to the pre-ictal baseline. (A) Percentage of channels detected as seizing. (B) Pairwise difference between the detected onset times relative to the reference threshold $\delta = 5$ used in the study. The solid line shows the median difference, the filled area corresponds to 10th to 90th percentile range. For the purpose of visualization, the difference is set as infinity for the channels that switched to non-seizing state. In both panels, the vertical line indicates the threshold used in the study.

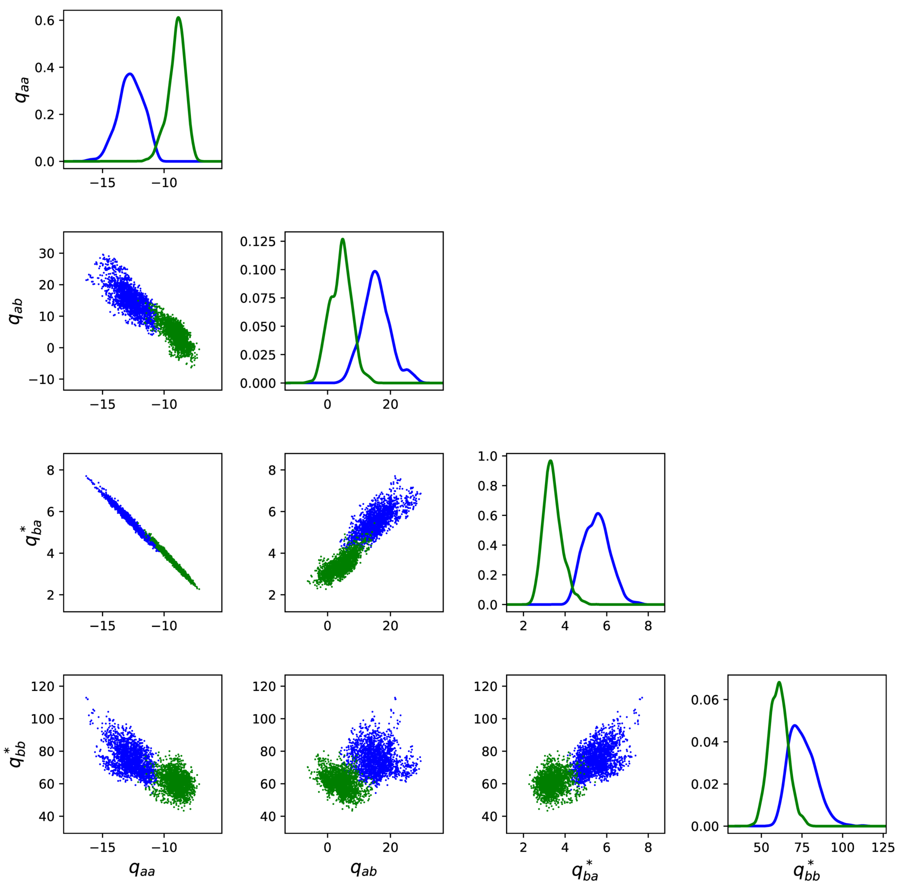

Fig 6. Posterior distribution of the hyperparameters. The diagonal panels show the kernel density estimation of the posterior distributions, the offdiagonal panels show the pair plots of the hyperparameters. The two colors represent two different data folds.

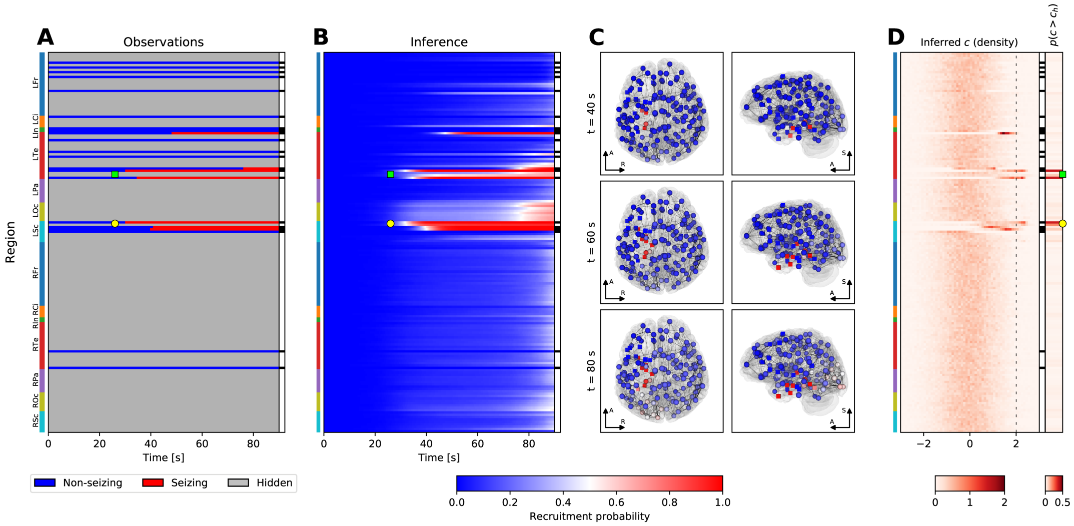

Fig 7. Example of the inference results on a seizure from subject 17. The layout of the figure is the same as in Fig 5 in the main text. The seizure is observed to start in the left anterior hippocampus (yellow circle, panels A, B) and left rhinal cortex and collateral sulcus (green square, panels A, B), and then it spreads to left amygdala and thalamus, left temporal pole, and eventually to left occipito-temporal sulcus. The seizure activity is inferred to remain spatially restricted, only the possible late recruitment of the left occipital lobe is inferred in addition to the observations. The early seizing regions are also inferred as being epileptogenic (yellow circle and green square, panel D).

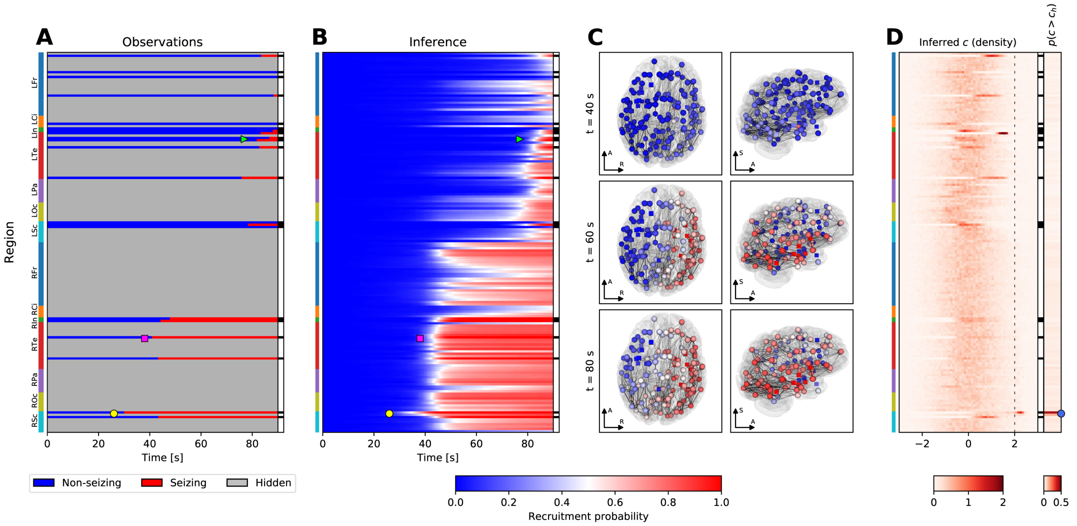

Fig 8. Example of the inference results on a seizure from subject 1. The layout of the figure is the same as in Fig 5 in the main text. From the observations the method infers that the seizure starts in the anterior part of the right hippocampus (yellow circle, panels A, B) before a large portion of the right hemisphere is recruited (magenta square, panels A, B), and eventually also some regions in the left hemisphere (light green triangle, panels A, B). The anterior part of the right hippocampus is strongly inferred to be the epileptogenic zone; the inference also points to the strongly connected posterior part of the right hippocampus (blue circle, panel D).

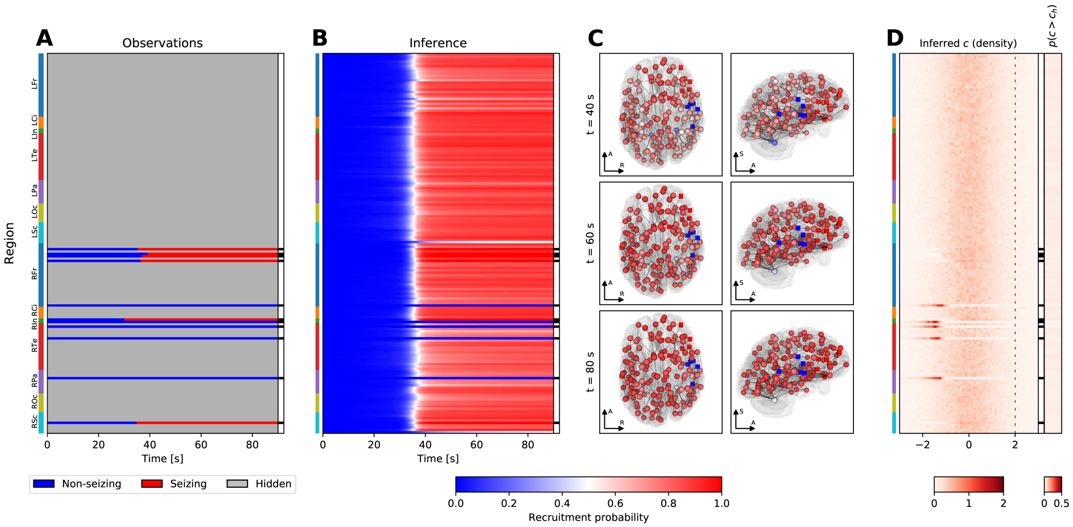

Fig 9. Example of the inference results on a seizure from subject 36. The layout of the figure is the same as in Fig 5 in the main text. This example shows a possible failure of the model, with a pattern repeatedly occurring among the results. Even though several stable regions are observed during the seizure, the method infers almost simultaneous recruitment of the majority of brain regions. The only regions not recruited are the observed stable nodes, and the left and right cerebella which are in the model generally very stable regions due to the low volumetric density of afferent projections obtained from diffusion-weighted imaging. None of the regions is identified as more epileptogenic than others; in cases of simulateneous recruitment it is difficult to infer which region is leading the seizure activity and which regions are only following.

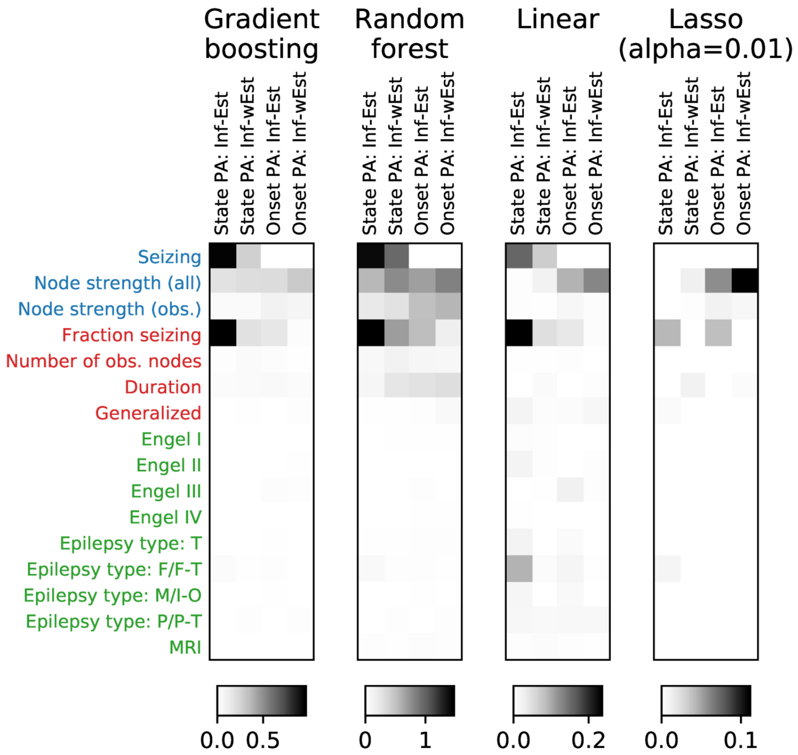

Fig 10. Feature importances from Fig 6B in the main text, calculated with four different methods: gradient boosting regression (shown in the main text), random forest regression, linear regression, and linear regression with lasso regularization ($\alpha = 0.01$).

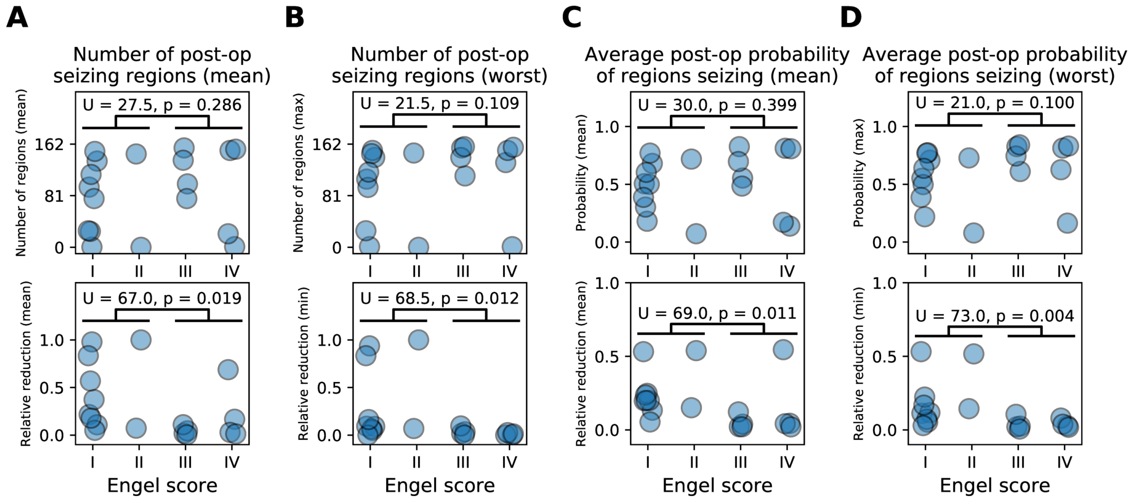

Fig 11. Extended analysis of the virtual resection results from Fig 7 in the main text. The columns show, for each patient with known surgery outcome, number of post-operative seizing regions after the virtual surgery (panels A and B) and the inferred post-operative probability that a region is seizing, averaged across all brain regions (panels C and D). When multiple seizures are available for a single patient, either the mean value (panels A and C) or the worst value (panels B and D) is shown. Top panels show the absolute measure (i.e, number of regions or the probability; higher is worse), lower panels show the relative reduction (higher is better). The text shows the Mann-Whitney statistic between patients with Engel score I and II and those with scores III and IV. Column A corresponds to Fig 7D in the main text.

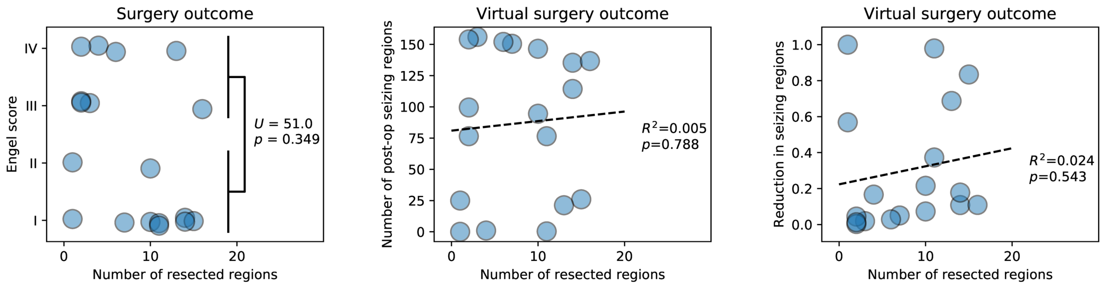

Fig 12. Dependency of the real and virtual surgery outcome from Fig 7 in the main text on the resection size. Real surgery outcome is quantified with the Engel score, the virtual surgery outcome with the post-operative number of seizing regions and with the reduction of seizing regions as on Fig 7 in the main text. The inset text show the result of statistical analysis: Mann-Whitney test between Engel I, II and III, IV patients for the real surgery outcome, and linear regression for the virtual surgery outcomes. Neither the real nor the virtual surgery outcome show strong dependency on the number of resected regions.

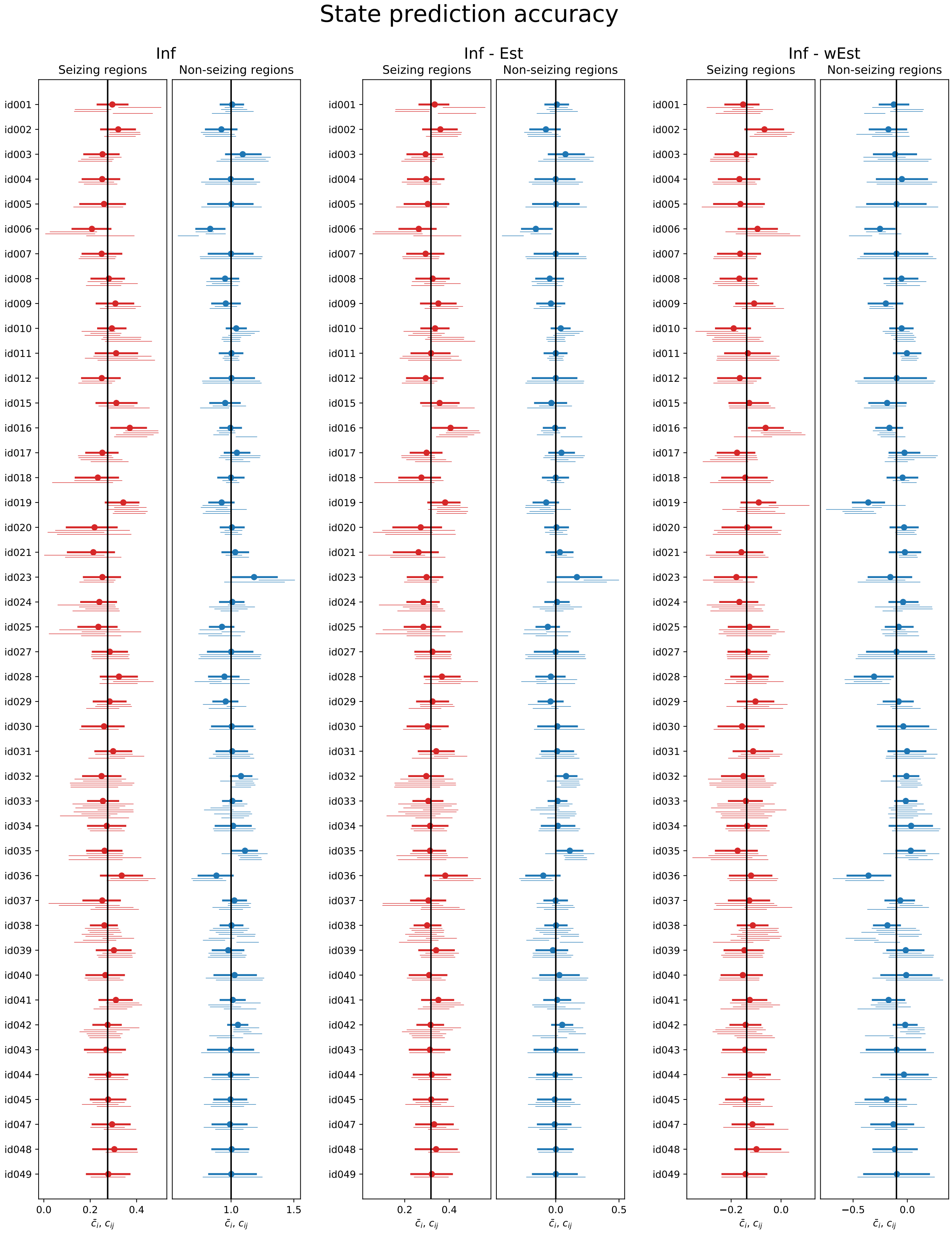

Fig 13. Multi-level analysis of state prediction accuracy. Full caption on the following page.

Fig 13. Multi-level analysis of state prediction accuracy. The plots show 95% confidence intervals for the intercepts $\bar{c}_i$ at the subject level (thick lines) and the intercepts c_{ij} at the seizure level (thin lines). Solid points show the median value. The columns show the results for the accuracy of inference (Inf), difference between the accuracies of the inference and the unweighted estimate (Inf - Est), and the difference between the accuracies of the inference and the weighted estimate (Inf - wEst), all separately for the seizing and non-seizing regions. Vertical line shows the mean of the global intercept $\bar{\bar{c}}$. In all panels, higher values mean better performance of the inference method, either absolute or compared to the estimates. Note that since the accuracy of the unweighted estimate depends linearly on the fraction of seizing regions, which is accounted for in the multi-level model, the results in the first two columns differ only by a constant.

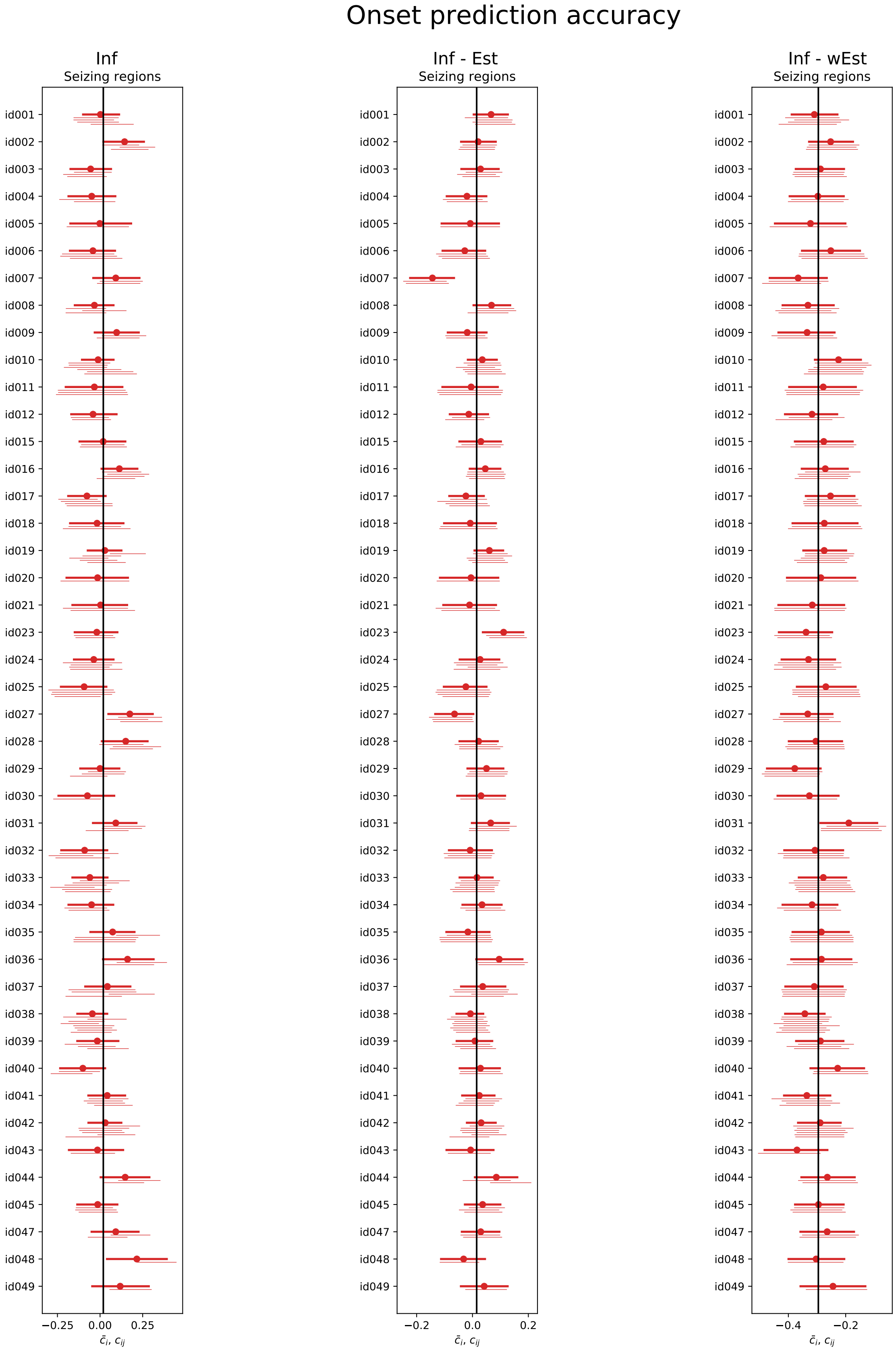

Fig 14. Multi-level analysis of onset prediction accuracy. Layout same as in Fig 13

Table 1. (Caption on the following page)

Operation	Name(s) of the input region(s)	Name(s) of the output region(s)	Options
merge	ctx_%h_G_and_S_frontomargin, ctx_%h_G_and_S_transv_frontopol	%H-Frontal-pole	
merge	ctx_%h_G_orbital, ctx_%h_S_orbital_lateral, ctx_%h_S_orbital-H_Shaped	%H-Orbito-frontal-cortex	
merge	ctx_%h_G_rectus, ctx_%h_S_suborbital, ctx_%h_S_orbital_med-olfact	%H-Gyrus-rectus	
merge	ctx_%h_G_front_inf-Orbital, ctx_%h_Lat_Fis-ant-Horizont	%H-F3-Pars-Orbitalis	
merge	ctx_%h_G_front_inf-Triangul, ctx_%h_Lat_Fis-ant-Vertical	%H-F3-Pars-triangularis	
merge	ctx_%h_G_temp_sup-G_T_transv, ctx_%h_S_temporal_transverse	%H-Gyrus-of-Heschl	
merge	ctx_%h_Lat_Fis-post, ctx_%h_G_temp_sup-Plan_tempo	%H-T1-planum-temporale	
merge	ctx_%h_G_occipital_sup, ctx_%h_S_oc_sup_and_transversal	%H-O1	
merge	ctx_%h_G_precuneus, ctx_%h_S_subparietal	%H-Precuneus	
merge	ctx_%h_G_occipital_middle, ctx_%h_S_oc_middle_and_Lunatus	%H-O2	
rename	ctx_%h_G_front_inf-Opercular	%H-F3-pars-opercularis	
rename	ctx_%h_S_front_inf	%H-Inferior-frontal-sulcus	
rename	ctx_%h_S_front_middle	%H-Middle-frontal-sulcus	
rename	ctx_%h_G_subcallosal	%H-Subcallosal-area	
rename	ctx_%h_S_precentral-inf-part	%H-Precentral-sulcus-inferior-part	
rename	ctx_%h_S_precentral-sup-part	%H-Precentral-sulcus-superior-part	
rename	ctx_%h_G_and_S_paracentral	%H-Paracentral-lobule	
rename	ctx_%h_Pole_temporal	%H-Temporal-pole	
rename	ctx_%h_G_temp_sup-Plan_polar	%H-T1-planum-polare	
rename	ctx_%h_G_oc-temp_lat-fusifor	%H-Fusiform-gyrus	
rename	ctx_%h_G_postcentral	%H-Postcentral-gyrus	
rename	ctx_%h_S_postcentral	%H-Postcentral-sulcus	
rename	ctx_%h_G_parietal_sup	%H-Superior-parietal-lobule-P1	
rename	ctx_%h_G_pariet_inf-Angular	%H-Angular-gyrus	
rename	ctx_%h_S_intrapariet_and_P_trans	%H-Intraparietal-sulcus	
rename	ctx_%h_S_cingul-Marginalis	%H-Marginal-branch-of-the-cingulate-sulcus	
rename	ctx_%h_S_parieto_occipital	%H-Parieto-occipital-sulcus	
rename	ctx_%h_S_occipital_ant	%H-Anterior-occipital-sulcus-and-preoccipital-notch	
rename	ctx_%h_G_and_S_occipital_inf	%H-O3	
rename	ctx_%h_Pole_occipital	%H-Occipital-pole	
rename	ctx_%h_G_oc-temp_med-Lingual	%H-Lingual-gyrus	
rename	ctx_%h_S_calcarine	%H-Calcarine-sulcus	
rename	ctx_%h_G_cuneus	%H-Cuneus	
split	ctx_%h_G_front_middle	%H-F2-rostral, %H-F2-caudal	
split	ctx_%h_S_front_sup	%H-SFS-rostral, %H-SFS-caudal	
split	ctx_%h_G_and_S_subcentral	%H-Central-operculum, %H-Parietal-operculum	
split	ctx_%h_G_temp_sup-Lateral	%H-T1-lateral-anterior, %H-T1-lateral-posterior	
split	ctx_%h_S_temporal_sup	%H-STS-anterior, %H-STS-posterior	
split	ctx_%h_S_temporal_inf	%H-ITS-anterior, %H-ITS-posterior	
split	ctx_%h_G_temporal_middle	%H-T2-anterior, %H-T2-posterior	
split	ctx_%h_G_temporal_inf	%H-T3-anterior, %H-T3-posterior	
split-mes	ctx_%h_G_front_sup	%1, %2	
split	%1	%H-F1-mesial-prefrontal, %H-PreSMA, %H-SMA	ratios: 2, 1, 3
split	%2	%H-F1-lateral-prefrontal, %H-F1-lateral-premotor	
split	ctx_%h_G_precentral	%H-Precentral-gyrus-head-face, %H-Precentral-gyrus-upper-limb	ratios: 2, 1
split	ctx_%h_S_central	%H-Central-sulcus-head-face, %H-Central-sulcus-upper-limb	ratios: 2, 1
split	ctx_%h_S_oc-temp_med_and_Lingual	%H-Collateral-sulcus, %1	
merge	%1, ctx_%h_S_collat_transv_post	%H-Lingual-sulcus	
split	ctx_%h_G_oc-temp_med-Parahip	%1, %H-Parahippocampal-cortex	
split	ctx_%h_S_collat_transv_ant	%2, %3	
merge	%1, %2	%H-Rhinal-cortex	
merge	%3, ctx_%h_S_oc-temp_lat	%H-Occipito-temporal-sulcus	
split	ctx_%h_G_pariet_inf-Supramar	%H-Supramarginal-anterior, %1	
merge	%1, ctx_%h_S_interm_prim-Jensen	%H-Supramarginal-posterior	
split-to-nl	ctx_%h_S_pericallosal	ctx_%h_G_and_S_cingul-Ant, ctx_%h_G_and_S_cingul-Mid-Ant, ctx_%h_G_and_S_cingul-Mid-Post, ctx_%h_G_cingul-Post-dorsal, ctx_%h_G_cingul-Post-ventral	
rename	ctx_%h_G_and_S_cingul-Ant	%H-Anterior-cingulate-cortex	
rename	ctx_%h_G_and_S_cingul-Mid-Ant	%H-Middle-cingulate-cortex-anterior-part	
rename	ctx_%h_G_and_S_cingul-Mid-Post	%H-Middle-cingulate-cortex-posterior-part	
rename	ctx_%h_G_cingul-Post-dorsal	%H-Posterior-cingulate-cortex-dorsal	
rename	ctx_%h_G_cingul-Post-ventral	%H-Posterior-cingulate-cortex-retrosplenial-gyrus	
split-to	ctx_%h_S_circular_insula_sup	ctx_%h_G_insular_short, ctx_%h_G_Ins_lg_and_S_cent_ins	
merge	ctx_%h_S_circular_insula_ant, ctx_%h_G_insular_short	%H-Insula-gyri-brevi	
merge	ctx_%h_G_Ins_lg_and_S_cent_ins, ctx_%h_S_circular_insula_inf	%H-Insula-gyri-longi	
split	%H-Hippocampus	%H-Hippocampus-anterior, %H-Hippocampus-posterior	
rename	%H-Cerebellum-Cortex	%H-Cerebellar-cortex	
rename	%H-Thalamus-Proper	%H-Thalamus	
rename	%H-Caudate	%H-Caudate-nucleus	
rename	%H-Accumbens-area	%H-Nucleus-accumbens	

Table 1. List of operations to create the custom brain parcellation. The operations are: `merge` - merges two or more regions together; `rename` - renames a region; `split` - splits one region into multiple using a linear PCA projection of the coordinates; `split-to` - splits one region into multiple using a linear projection and merges the created regions with the specified existing ones; `split-to-nl` - splits one region into multiple using a nonlinear Isomap projection and merges the created regions with the specified existing ones; `split-mes` - splits the region into the mesial wall and the remaining part, using a criterion of the mesial-lateral component of the unit normal vector of the inflated cortical surface being equal to -0.5. Wildcards %h and %H stand for the hemisphere name in the format 'lh'/'rh' and 'Left'/'Right' respectively, and numerical wildcards %1, %2, %3 stand for temporary regions which are then replaced by following operations. If ratios are given, the split is into non-equal sized region as determined by the ratios, otherwise the region is split into equally sized regions. For the cortical regions, the operations are performed on the triangulated cortical surface, while for the subcortical regions the operations are performed on the voxels.

Table 2. List of brain regions in the custom atlas.

Anatomical grouping	Index	Name	Index	Name
Frontal lobe	1	Left-Frontal-pole	82	Right-Frontal-pole
Frontal lobe	2	Left-Orbito-frontal-cortex	83	Right-Orbito-frontal-cortex
Frontal lobe	3	Left-Gyrus-rectus	84	Right-Gyrus-rectus
Frontal lobe	4	Left-F3-Pars-Orbitalis	85	Right-F3-Pars-Orbitalis
Frontal lobe	5	Left-F3-Pars-triangularis	86	Right-F3-Pars-triangularis
Frontal lobe	6	Left-F3-pars-opercularis	87	Right-F3-pars-opercularis
Frontal lobe	7	Left-Inferior-frontal-sulcus	88	Right-Inferior-frontal-sulcus
Frontal lobe	8	Left-F2-rostral	89	Right-F2-rostral
Frontal lobe	9	Left-F2-caudal	90	Right-F2-caudal
Frontal lobe	10	Left-Middle-frontal-sulcus	91	Right-Middle-frontal-sulcus
Frontal lobe	11	Left-SFS-rostral	92	Right-SFS-rostral
Frontal lobe	12	Left-SFS-caudal	93	Right-SFS-caudal
Frontal lobe	13	Left-F1-mesial-prefrontal	94	Right-F1-mesial-prefrontal
Frontal lobe	14	Left-PreSMA	95	Right-PreSMA
Frontal lobe	15	Left-SMA	96	Right-SMA
Frontal lobe	16	Left-F1-lateral-prefrontal	97	Right-F1-lateral-prefrontal
Frontal lobe	17	Left-F1-lateral-premotor	98	Right-F1-lateral-premotor
Frontal lobe	18	Left-Subcallosal-area	99	Right-Subcallosal-area
Frontal lobe	19	Left-Precentral-sulcus-inferior-part	100	Right-Precentral-sulcus-inferior-part
Frontal lobe	20	Left-Precentral-sulcus-superior-part	101	Right-Precentral-sulcus-superior-part
Frontal lobe	21	Left-Precentral-gyrus-head-face	102	Right-Precentral-gyrus-head-face
Frontal lobe	22	Left-Precentral-gyrus-upper-limb	103	Right-Precentral-gyrus-upper-limb
Frontal lobe	23	Left-Central-sulcus-head-face	104	Right-Central-sulcus-head-face
Frontal lobe	24	Left-Central-sulcus-upper-limb	105	Right-Central-sulcus-upper-limb
Frontal lobe	25	Left-Paracentral-lobule	106	Right-Paracentral-lobule
Frontal lobe	26	Left-Central-operculum	107	Right-Central-operculum
Frontal lobe	27	Left-Parietal-operculum	108	Right-Parietal-operculum
Cingulate cortex	28	Left-Anterior-cingulate-cortex	109	Right-Anterior-cingulate-cortex
Cingulate cortex	29	Left-Middle-cingulate-cortex-anterior-part	110	Right-Middle-cingulate-cortex-anterior-part
Cingulate cortex	30	Left-Middle-cingulate-cortex-posterior-part	111	Right-Middle-cingulate-cortex-posterior-part
Cingulate cortex	31	Left-Posterior-cingulate-cortex-dorsal	112	Right-Posterior-cingulate-cortex-dorsal
Cingulate cortex	32	Left-Posterior-cingulate-cortex-retrosplenial-gyrus	113	Right-Posterior-cingulate-cortex-retrosplenial-gyrus
Insula	33	Left-Insula-gyri-brevi	114	Right-Insula-gyri-brevi
Insula	34	Left-Insula-gyri-longi	115	Right-Insula-gyri-longi
Temporal lobe	35	Left-Temporal-pole	116	Right-Temporal-pole
Temporal lobe	36	Left-T1-planum-polare	117	Right-T1-planum-polare
Temporal lobe	37	Left-Gyrus-of-Heschl	118	Right-Gyrus-of-Heschl
Temporal lobe	38	Left-T1-planum-temporale	119	Right-T1-planum-temporale
Temporal lobe	39	Left-T1-lateral-anterior	120	Right-T1-lateral-anterior
Temporal lobe	40	Left-T1-lateral-posterior	121	Right-T1-lateral-posterior
Temporal lobe	41	Left-STS-anterior	122	Right-STS-anterior
Temporal lobe	42	Left-STS-posterior	123	Right-STS-posterior
Temporal lobe	43	Left-ITS-anterior	124	Right-ITS-anterior
Temporal lobe	44	Left-ITS-posterior	125	Right-ITS-posterior
Temporal lobe	45	Left-T2-anterior	126	Right-T2-anterior
Temporal lobe	46	Left-T2-posterior	127	Right-T2-posterior
Temporal lobe	47	Left-T3-anterior	128	Right-T3-anterior
Temporal lobe	48	Left-T3-posterior	129	Right-T3-posterior
Temporal lobe	49	Left-Fusiform-gyrus	130	Right-Fusiform-gyrus
Temporal lobe	50	Left-Occipito-temporal-sulcus	131	Right-Occipito-temporal-sulcus
Temporal lobe	51	Left-Collateral-sulcus	132	Right-Collateral-sulcus
Temporal lobe	52	Left-Lingual-sulcus	133	Right-Lingual-sulcus
Temporal lobe	53	Left-Parahippocampal-cortex	134	Right-Parahippocampal-cortex
Temporal lobe	54	Left-Rhinal-cortex	135	Right-Rhinal-cortex
Parietal lobe	55	Left-Postcentral-gyrus	136	Right-Postcentral-gyrus
Parietal lobe	56	Left-Postcentral-sulcus	137	Right-Postcentral-sulcus
Parietal lobe	57	Left-Superior-parietal-lobule-P1	138	Right-Superior-parietal-lobule-P1
Parietal lobe	58	Left-Supramarginal-anterior	139	Right-Supramarginal-anterior
Parietal lobe	59	Left-Supramarginal-posterior	140	Right-Supramarginal-posterior
Parietal lobe	60	Left-Angular-gyrus	141	Right-Angular-gyrus
Parietal lobe	61	Left-Intraparietal-sulcus	142	Right-Intraparietal-sulcus
Parietal lobe	62	Left-Precuneus	143	Right-Precuneus
Parietal lobe	63	Left-Marginal-branch-of-the-cingulate-sulcus	144	Right-Marginal-branch-of-the-cingulate-sulcus
Parietal lobe	64	Left-Parieto-occipital-sulcus	145	Right-Parieto-occipital-sulcus
Occipital lobe	65	Left-Anterior-occipital-sulcus-and-preoccipital-notch	146	Right-Anterior-occipital-sulcus-and-preoccipital-notch
Occipital lobe	66	Left-O3	147	Right-O3
Occipital lobe	67	Left-O2	148	Right-O2
Occipital lobe	68	Left-O1	149	Right-O1
Occipital lobe	69	Left-Occipital-pole	150	Right-Occipital-pole
Occipital lobe	70	Left-Lingual-gyrus	151	Right-Lingual-gyrus
Occipital lobe	71	Left-Calcarine-sulcus	152	Right-Calcarine-sulcus
Occipital lobe	72	Left-Cuneus	153	Right-Cuneus
Subcortical regions	73	Left-Hippocampus-anterior	154	Right-Hippocampus-anterior
Subcortical regions	74	Left-Hippocampus-posterior	155	Right-Hippocampus-posterior
Subcortical regions	75	Left-Amygdala	156	Right-Amygdala
Subcortical regions	76	Left-Thalamus	157	Right-Thalamus
Subcortical regions	77	Left-Caudate-nucleus	158	Right-Caudate-nucleus
Subcortical regions	78	Left-Putamen	159	Right-Putamen
Subcortical regions	79	Left-Pallidum	160	Right-Pallidum
Subcortical regions	80	Left-Nucleus-accumbens	161	Right-Nucleus-accumbens
Subcortical regions	81	Left-Cerebellar-cortex	162	Right-Cerebellar-cortex

Table 3. Patient table. Abbreviation in bracket in the Epilepsy type column indicates the simplified epilepsy type used in the statistical analysis (T, Temporal; P/P-T, Posterior/Postero-temporal; F/F-T, Frontal/Fronto-temporal; M/I-O, Motor/Insulo-opercular). Other abbreviations: AVM, arteriovenous malformation; DNET, dysembryoplastic neuroepithelial tumor; FCD, focal cortical dyplasia; HH, hypothalamic hamartoma; L, left; NA, not applicable; PMG, polymicrogyria; PNH, periventricular nodular heterotopia; R, right; SG, secondarily generalized. The column "Number of seizures" shows the number of recorded seizures longer than 30 seconds used in this study.

Patient	Gender	Age at epilepsy onset (y)	Epilepsy duration (y)	Epilepsy type	MRI	Histopathology	Side	Engel score	Post-op MRI	Number of seizures	Number of SG seizures
1	F	31	3	Temporo-insular (T)	Normal	Hippocampal sclerosis	R	I	Y	4	1
2	F	19	10	Temporo-occipital (P/P-T)	L temporo-occipital PNH	NA	L	NA	N	3	0
3	M	23	13	Temporo-frontal (F/F-T)	R temporo-occipital scar	FCD1a	R	I	Y	3	3
4	F	23	3	Temporal (T)	R temporal mesial ganglioglioma	Ganglioglioma	R	I	Y	2	0
5	M	0.3	21	Postcentral - superior parietal (M/I-O)	L postcentral-parietal gyration asymmetry	NA	L	NA	N	1	1
6	M	45	14	Fronto-temporal (F/F-T)	Normal	NA	L	NA	N	3	0
7	M	55	5	Temporal (T)	Normal	Slight gliosis	R>L	III	Y	2	2
8	F	38	8	Temporal (T)	L amygdala enlargement	Slight gliosis	L	III	N	3	2
9	F	11	34	Bifocal: parietal mesial & temporo-basal	Unknown R parietal lesion	Rosenthal fibers; slight gliosis	R	III	Y	2	0
10	F	27	18	Temporal (T)	L hippocampal sclerosis	Hippocampal sclerosis	L	I	N	6	0
11	F	27	14	Frontal (F/F-T)	L frontal scar (abcess)	Gliosis	L	IV	Y	3	0
12	F	19	9	Bilateral temporo-frontal (F/F-T)	Bilateral hippocampal & amygdala T2-hypersignal	NA	R&L	NA	N	2	2
13	M	2	17	Frontal (F/F-T)	Normal	Slight gliosis	L	I	N	2	0
14	F	5	18	Premotor (M/I-O)	Normal	FCD2b	L	I	Y	0	0
15	M	8	33	Temporal (T)	R temporal PMG & multiple PNH	NA	R	NA	N	2	1
16	M	6	23	Temporo-operculo-fronto-parietal	R temporo-parieto-insular & L temporo-parietal necrosis	NA	R>L	NA	N	4	1
17	M	5	21	Temporal (T)	L temporo-polar hypothrophy and hippocampal sclerosis	Hippocampal sclerosis; gliosis	L	I	Y	4	1
18	M	2	22	Parieto-temporal (P/P-T)	L Parieto-occipital necrosis (perinatal anoxy)	NA	L	NA	N	2	2
19	M	29	15	Temporo-insular (T)	Normal	NA	L>R	NA	N	5	0
20	F	17	10	Temporal (T)	Normal	Hippocampal sclerosis	R	I	Y	3	0
21	F	9	14	Occipital (P/P-T)	Normal	FCD1c	L	II	Y	2	0
22	F	7	23	Parietal (P/P-T)	L parietal FCD	FCD2b	L	I	Y	0	0
23	M	35	28	Temporal (T)	Normal	NA	L	NA	N	2	2
24	M	14	15	Temporal (T)	Normal	NA	R	NA	N	4	1
25	M	7	35	Insular (M/I-O)	Normal	NA	L	I	Y	4	0
26	F	4	24	Occipital (P/P-T)	PNH	NA	R	NA	N	0	0

Table 3. Patient table (continued).

Patient	Gender	Age at epilepsy onset (y)	Epilepsy duration (y)	Epilepsy type	MRI	Histopathology	Side	Engel score	Post-op MRI	Number of seizures	Number of SG seizures
27	M	17	12	Frontal (F/F-T)	R prefrontal gliotic scar (AVM)	Gliosis	R>L	II	N	3	3
28	F	8	14	Temporo-frontal (F/F-T)	Anterior temporal necrosis	Gliosis	R	III	Y	3	0
29	F	21	9	Bilateral temporal (T)	Bilateral posterior PNH	NA	R>L	NA	N	3	0
30	M	11	45	Temporo-frontal (F/F-T)	R Frontal FCD	FCD 2	R	I	Y	1	0
31	F	20	18	Occipital (P/P-T)	Normal	NA	R	NA	N	3	0
32	F	15	21	Bilateral temporal with HH (T)	L HH	NA	R&L	NA	N	5	0
33	F	18	5	Temporo-parieto-opercular (P/P-T)	Normal	Hippocampal sclerosis	R	IV	Y	9	0
34	F	33	8	Temporal (T)	Multiple R temporo-parietal PNH & temporal PMG	NA	R	NA	N	2	2
35	M	4	27	Bilateral occipito-temporal (P/P-T)	R occipital mesial FCD	NA	R&L	NA	N	4	2
36	F	8	13	Temporo-insular (T)	R temporal anterior resection cavity	Gliosis	R	IV	Y	3	0
37	M	28	5	Temporal mesial (T)	R temporo-polar & amygdala FCD, L post-chiasmal pilocytic astrocyrtoma	FCD 2b	R	III	Y	4	2
38	M	11	22	Bilateral temporal (T)	Normal	NA	L&R	NA	N	8	2
39	M	40	4	Temporo-frontal (F/F-T)	R fronto-temporal necrosis (gunshot injury)	Gliosis	R	I	Y	3	0
40	F	16	19	Temporal mesial (T)	Hippocampal sclerosis	Hippocampal sclerosis	L	II	Y	2	0
41	M	0.7	26	Bilateral, temporal predominant	R perisylvian necrosis (perinatal stroke)	NA	R>L	NA	N	4	1
42	F	9	19	Temporal mesial (T)	Bilateral hippocampal sclerosis	NA	L	NA	N	6	0
43	F	7	16	Premotor (M/I-O)	R precentral FCD	NA	R	NA	N	1	1
44	M	0.5	39	Multifocal: parieto-operculo-premotor; temporal mesial	L hippocampal & amygdala T2 hypersignal	NA	L	NA	N	2	0
45	F	24	17	Temporal mesial (T)	Normal	NA	L	IV	Y	3	0
46	M	1.5	31	Insulo-parieto-premotor (M/I-O)	Normal	NA	R	NA	N	0	0
47	M	16	13	Bilateral frontal (F/F-T)	Normal	NA	R&L	NA	N	2	2
48	F	15	7	Premotor (M/I-O)	R parietal DNET	NA	R	NA	N	1	1
49	F	1	21	Motor-opercular (M/I-O)	R fronto-opercular resection cavity	NA	R	NA	N	1	1
50	M	14	21	Motor-premotor (M/I-O)	L insulo-opercular necrosis (stroke)	NA	L	I	Y	0	0
