# Supplementary material for: Data-driven method to infer the seizure propagation patterns in an epileptic brain from intracranial electroencephalography
Source: PLoS Comput Biol. 2021 Feb 17;17(2):e1008689. doi: 10.1371/journal.pcbi.1008689 (PMC7920393; doi:10.1371/journal.pcbi.1008689)
Supplement: Attachment — Submitted filename: ResponseLetter_v1.1.pdf [file pcbi.1008689.s002.pdf]

Manuscript PCOMPBIOL-D-20-01581
Response to reviewers

Dear Dr Marinazzo,

We submit the revised version of our manuscript titled "Data-driven method to infer the seizure propagation patterns in an epileptic brain from intracranial electroencephalography". We would like to thank the reviewers for careful reading, constructive criticism, and useful suggestions. We have made numerous changes to address the points raised, leading, as we believe, to substantially improved manuscript. We reply to the comments individually below. Attached is the revised manuscript, and a version with the changes highlighted.

We employ the following conventions in the replies:

- Original reviewers' comments are in black.
- *Our replies are in green italics.*
- ***References to sections, figures, or tables in revised manuscript are in bold green italics.***
- *Full or partial elements of the revised text are in red italics.*

For easier orientation and referencing, we have added consistent numbering to the original comments.

Reviewer #1: In this article, the authors develop a computational model of macroscopic brain activity. The model combines a patient's structural connectome with observed brain activity from intracranial electrodes to infer brain electrical activity at unobserved brain regions during seizures. The presentation is clear, and the approach compelling. As the authors admit, the clinical results are mildly underwhelming (eg the model does not outperform empirical observations, the resection results are rather weak). But the framework is excellent. The model is simple and data-driven. The clear presentation of model assumptions is excellent. I believe this work is compelling and important to report to the scientific community. Please find below my comments, which I hope the authors find helpful.

Major Comments

R1.1. Would the authors please provide the code used to (i) simulate an instance of the model, (ii) infer the hidden activity traces from the simulated data. For example, code to generate Figure 2 would be extremely helpful to enhance reproducibility of the work. Providing the code on a public repository (e.g., GitHub) and/or with the publication would be appreciated.

The code is made available at github.com/ins-amu/ddmisp. In particular, the code to generate Figure 2 is under ddmisp/ddmisp/notebooks/Fig_SimulatedSeizure.ipynb, and the statistical models used for the inference are under ddmisp/ddmisp/stan/.stan.*

R1.2. Do the results in Figure 3 depend on the method to detect the onset time? The method requires choosing a threshold and assigning a binary category to each channel. Would a different threshold impact the results?

*The influence of the threshold on the detected onset times is now evaluated in the newly added **Fig. 5 in Supplementary information**, and described in the main text:*
Inevitably, the results of the onset time detection (and of the subsequent analysis) depend on the parameters of the detection method, most notably on the signal power threshold that determines the seizing state. On Fig. 5 in S1 Text we analyze the influence of this choice. Unfortunately, the results are smoothly dependent on the threshold value, and no clear value of the threshold presents itself via a plateau or a sharp drop. We continue with the (to an extent arbitrarily) chosen threshold $\delta = 5$, however, we return to the question of the binary distinction of seizing and non-seizing state in Discussion.

*We then discuss the binary state in **Discussion/Assumptions of the method/Binary healthy/seizure state**. The influence of the threshold on results further in the workflow (LOO validation results, in-silico resection results) is not analyzed due to high computational costs of the inference procedure.*

R1.3. The measures reported in Figure 4 seem quite complicated. Can the resection results be explored in another way, without performing a virtual surgery? For example, could the authors work directly with the patient clinical results, and compare excitability in a patient's resected tissue vs in non-resected tissue, with the hypothesis that excitability is higher in resected tissue when Engel I or II? Or something similar? Maybe this analysis is done in Figure 7E, but that was difficult to understand.

We assume that the comment intended to reference ***Fig.*** *7 instead of* ***Fig. 4****. The analysis analogous to the requested one is indeed performed in* ***Fig.*** *7E, where the values of precision and recall are presented for all Engel classes and for different thresholds. Precision-recall analysis is a standard way to assess the relevance in binary classification problems. The two values quantify whether the highly excitable nodes are located in the resected areas (precision) and whether the resected areas have elevated excitability (recall). We have extended its description and motivation in the main text so its purpose is clearer:*

Further insight can be gained by a direct analysis of the excitability results without relying on the virtual resection. We have evaluated the match between the regions that were identified as epileptogenic by the model and the regions that were surgically resected (Fig. 7E). If the model is to be useful, we expect that these should overlap more in the subjects where surgery was successful, and less where the surgery failed. To analyze the match, we plot the precision-recall curves. Precision-recall analysis is suitable for binary classification tasks on unbalanced data sets where number of true negatives (that is, non-resected regions not identifed as epileptogenic) outweights the number of positives (Saito and Rehmsmeier, 2015). In this context, the precision expresses how many of the predicted epileptogenic regions were resected, and the recall expresses how many of the resected regions were predicted to be epileptogenic. These values are computed for varying threshold on what constitutes a highly epileptogenic region. A perfect match would result in a curve passing through top-right corner, while bottom-left corner indicates a mismatch. In the results we indeed see better performance among the Engel I and Engel II classes than Engel III and Engel IV classes, even if the performance does not follow the surgery outcome precisely: our results show the order of Engel classes II > I > IV > III.

R1.4. The guiding principle of the method is simplicity over complexity (line 311). However, the clinical results of the model are weak. So, the argument could be made that the model is too simple to be useful. A stronger result in (3) would help counter this argument.

Actually we are of the opinion that building simple models and analyzing when, how, and why they fail (or succeed) is a necessary step towards the development of models useful in research and clinical practice. Especially the failures are interesting, as the data features used here are entirely focused on the spatiotemporal seizure pattern, ignoring the features of the offset pattern. The latter, however, are part of more complex epilepsy models, such as the Epileptor (phenomenological, Jirsa et al 2014) or Wendling et al (physiological, Wendling et al 2002). The deeper understanding will lead to better clinical tools and practice. This is where we see the value. In this spirit, more detailed analysis of the validation results is now presented in ***Fig. 6.*** *In particular* ***Fig. 6C*** *analyzes which factors contribute to the predictive performance of the model.*

R1.5. Could the authors comment on the applicability of this approach to other domains, eg, non-seizure activity, scalp EEG?

*We have added a paragraph discussing the use of scalp EEG with the method (****Discussion/Towards non-invasive EEG?****):*
In the present work we use intracranial EEG to inform us about the seizure evolution. Given the medical risk of the invasive method for the patient (Jayakar et al., 2016), the appeal of exploiting non-invasive EEG recordings instead is obvious. Our model operates in the source space, that is on the level of brain regions, and thus a sensor-to-source projection is needed prior to running the method. With intracranial EEG we employed a straightforward distance-

based mapping. The source inversion of scalp EEG however poses much larger challenge despite the advances in EEG source localization techniques and increased availability of high-density EEG (Michel and Brunet, 2019). In the field of epilepsy the focus remains on localizing either the interictal spikes, or, in case of ictal source inversion, on localizing the seizure onset zone using the recording from the seizure onset only (van Mierlo et al., 2020). Mapping the entire seizure propagation to source space, as our method would require, is a problem not sufficiently tackled yet. Exploiting the non-invasive data in the current framework thus awaits the development and validation of robust source inversion method for spatially extended and time-varying sources.

We refrain from commenting on the use of the method for non-seizure activity; the method was designed for analyzing the seizure activity, and we do not see other problems in neuroscience that would clearly call for the use of such method without extensive modifications.

Minor Comments

R1.6. Line 28-29. The two references refer to a simulation study and fMRI. Can the authors provide evidence for this claim for voltage activity? Or perhaps remove the work “strong” from the text.

We have updated the text:
In healthy brains there exists a link between the structural and functional connectivity obtained from resting state fMRI (Ghosh et al., 2008; Honey et al; 2009).

R1.7. Line 166-167, How to interpret R_hat and N_eff? Is N_eff = 30 good or bad? Can the authors provide 1 sentence definitions for each?

We have provided the better descriptions of the two indicators. The paragraph now reads:

The R^ value indicates how well the Markov chains mix, and loosely it is defined as the ratio of within-chain and between-chains parameter variance. The value of 1 indicates perfect mixing, and higher values point towards the chains not mixing well. Number of effective samples N_eff is the estimate of the number of independent samples with the same estimation power as the obtained autocorrelated samples. Here in some cases the R^ values lie slightly outside of the generally recommended range R^ < 1.1 indicating imperfect mixing of the MCMC chains; we consider it acceptable given the practical limitations of the required time for the computations and considering that the hyperparameter posterior distribution will be reduced to point estimate for the second step of fitting the individual seizures. For the same reason we also do not consider the low number of effective samples problematic.

R1.8. Line 182. Could the authors provide more explanation of Figure 4B in the main text? This Figure seems to be important, and the text in Results is maybe too concise.

The text was extended to better describe the figure. The full paragraph now reads:
If the model is sufficiently flexible and fitted well, it should be able to generate synthetic seizures resembling the real ones. Testing if it does is the goal of posterior predictive checks. We took the two hyperparameter sets obtained by fitting the two folds, we randomly drew the excitabilities from the prior standard normal distribution, and we simulated an ensemble of 137 seizures - same number as of the real seizures in the data set. We repeated this process

one hundred times to obtain hundred ensembles of simulated seizures for both folds and then we compared the statistical properties of the synthetic seizure ensembles with the ensemble of real seizures (Fig. 4B). We chose five statistics to evaluate the similarity between real and simulated seizures: Fraction of the seizing regions in one seizure, and standard deviation and 10th, 50th, and 90th percentile of the onset times of the seizing regions. For a well fitted and sufficiently flexible model, the statistics of the synthetic seizures should overlap with the statistics of the real seizures. Furthermore, the statistics of the simulations from two folds should overlap, the opposite would point to an overfitting to the subsampled data set. The results reveal that the two models from two folds indeed produce statistically similar seizures, indicating again that some common features of seizure dynamics were extracted from the data and the models were not overfitted. However the match with the real seizures is not ideal: the models generate less seizures with only few regions recruited, while overpredicting the number of seizures with majority (but not all) of the regions recruited (Fig. 4B, leftmost panel). That might indicate that the model is not sufficiently flexible to reliably reproduce the seizures that do not spread to the whole brain. Other panels in Fig. 4B nevertheless show that the statistics of the onset times of seizing regions are well reproduced: the model is sufficient in this aspect.

R1.9 Equation after line 523, Does the choice of function f matter? This choice seems rather complicated.

*The paragraph **Materials and Methods/Dynamical model of seizure propagation/Parameterization of the excitation function f_q** was revised and extended, and a sketch to illustrate the parameterization was added.*

R1.10. The authors might further motivate their model by citing the cellular automaton model of Traub in,
Traub, R. D., Duncan, R., Russell, A. J. C., Baldeweg, T., Tu, Y., Cunningham, M. O., & Whittington, M. A. (2010). Spatiotemporal patterns of electrocorticographic very fast oscillations (> 80 Hz) consistent with a network model based on electrical coupling between principal neurons. Epilepsia, 51(8), 1587–1597. http://doi.org/10.1111/j.1528-1167.2009.02420.

We now cite the work to provide better contextualization of our efforts:
As such, the model drops some complexity of the existing models while keeping the core elements of network models of seizure propagation: two possible states of a network node (either a healthy or a seizure state), the role of the network connections in seizure spread, and the role of heterogeneous node excitability. This state-based approach can be reminiscent of the cellular automatons, used also for epilepsy modeling (Traub et al., 2010).

Reviewer #2: In this paper, the authors introduce a novel method to infer the ictogenicity of brain regions not mapped by intracranial EEG which may aid clinicians in the decision-making process preceding epilepsy surgery. The paper is well written, and the method is interesting and offers significant potential for further investigations and improvements.

Major issues

R2.1. Despite the fact that the authors write that they considered 50 individuals, Table S3 seems to indicate that there were a number of them that did not have any recorded seizures,

making them presumably not usable in this study. I suggest that the authors make this clear or remove the individuals from Table S3 while correcting the numbers mentioned throughout the text. It would also be useful to clarify how many individuals were considered in each analysis upon applying their criteria.

*Even though some subjects did not have any recorded seizures that would satisfy the inclusion criteria, their structural data (connectomes) were used for the validation with the synthetic data (**Supplementary Information: Validation on synthetic data**). Therefore we keep their information in the table. However, clarification of this was included in **Materials and Methods/Patients and data acquisition**:*

Only the seizures with duration longer than 30 seconds were included in the analysis (see below for the reason why), and subclinical seizures were not considered. Apart from that, no other criteria for exclusion were applied. Five patients in the cohort did not have any seizures satisfying the duration criterion. Their structural connectomes, described in the following section, were constructed nevertheless, and they were used for generating and analyzing synthetic seizures (S1 Text/Validation on synthetic data). Exclusion of the short seizures left 45 patients for which at least one seizure was available.

*Furthermore, number of patients used in each analysis was added to figure captions where missing (**Fig. 6, Fig.** 7) and to the main text (**Results/Onset time detection and mapping, Results/Leave-One-Out cross-validation**, **Results/Validation against the resected regions**).*

R2.2a. I found Fig. 6C and 6F and the accompanying text (lines 228-236) somewhat misleading, because the authors choose to present an analysis for the apparent sole purpose of boosting their method, while choosing to present only as a supplementary material (Fig. S9) a comparison that is not as supportive. I would suggest to either move panels 6C and 6F to Fig. S9, or merge S9 to Fig. 6. I also suggest editing the text in lines 228-236 accordingly.

*We have substantially revised **Fig. 6** in order to include more extensive and more systematic analysis of the leave-one-out validation results. This new analysis presents an unbiased assessment of the strengths and weaknesses of the method. In particular, analysis of the state and onset prediction accuracies for both the unweighted and weighted estimate is now included in **Fig. 6**. Associated main text was also appropriately edited to accommodate the changes.*

R2.2b. Likewise, the statement in the abstract: "we demonstrate that the method can improve the predictions of the states of the unobserved regions compared to an empirical estimate" – this seems also somewhat misleading. While true, it conceals the fact that the method did not perform better than the ‘weighted estimate’.

(Nevertheless, I commend the authors for showing that a simple heuristic such as the ‘weighted estimate’ can produce results comparable to their method.)

The statement in the abstract was revised to the following:
Using a leave-one-out cross-validation scheme on a cohort of 45 patients we demonstrate that the method can improve the predictions of the states of the unobserved regions compared to an empirical estimate that does not use the structural information, yet it is on the same level as the estimate that takes the structure into account.

R2.2c. Also, in lines 224-225, the authors wrote: "The results demonstrate high predictive power of the inference method". It is unclear whether it is high, because it seems comparable to the 'baselines' introduced. I suggest that the authors introduce perhaps another baseline such as a random predictor or apply their method to surrogates with randomized distributions of seizing regions and onset times.

The statement was revised to avoid the qualification of 'high predictive power'.
The results demonstrate that the seizing/non-seizing state of a region can be predicted with a median state prediction accuracy 80.5%, and the onset time can be predicted with median onset prediction accuracy 29.9% (Fig. 6A). On group level, these values are however not better than those of both the unweighted and the weighted estimates (Fig. 6B).

R2.3a. I suggest that the authors add to Fig. 7 a ROC analysis (with AUC, sensitivity and specificity), as it is common in other studies of virtual resections.

The precision-recall (PR) analysis, instead of sensitivity-specificity analysis, was chosen intentionally. The PR analysis is better suited for imbalanced data sets, where the number of true negatives (in this context, non-resected nodes identified as non-epileptogenic) outweighs the number of positives. ROC plots are visually appealing, but maybe also misleading. For detailed analysis see:

Saito T, Rehmsmeier M (2015) The Precision-Recall Plot Is More Informative than the ROC Plot When Evaluating Binary Classifiers on Imbalanced Datasets. PLoS ONE 10(3): e0118432. https://doi.org/10.1371/journal.pone.0118432

We have explained this motivation in the main text and included this reference:
To analyze the match, we plot the precision-recall curves. Precision-recall analysis is suitable for binary classification tasks on unbalanced data sets where number of true negatives (that is, non-resected regions not identifed as epileptogenic) outweights the number of positives (Saito and Rehmsmeier, 2015). In this context, the precision expresses how many of the predicted epileptogenic regions were resected, and the recall expresses how many of the resected regions were predicted to be epileptogenic.

R2.3b. Also, why did the authors choose to look at the reduction of the number of seizing regions, rather than looking at the reduction of the overall excitability (which has the advantage of avoiding an arbitrary threshold)? One could expect that successful surgeries should have a higher reduction of the relative brain excitability compared to unsuccessful surgeries. I suppose both are reasonable options, and thus both could be assessed (e.g. one could be shown as a supplementary figure).

*The proposed analysis is now included in **Fig. 11 in Supplementary information**. The analysis shows the same qualitative results as the original one in **Fig.** 7.*

R2.3c. Also, it may be questionable whether using the average values across seizures is the best option to present these results. For example, one could argue that the minimum reduction across seizures may be a better indicator, because if different seizures are driven by different

mechanisms and hence providing different results, then one has to be as conservative as possible to make sure that all seizure generators are accounted for.

The proposed analysis was included in ***Fig. 11 in Supplementary information.*** *The analysis shows the same qualitative results as the original one in* ***Fig.*** *7.*

Minor issues

R2.4. Introduction, line 62, the authors wrote: "In this work we approach the problem of seizure propagation in a network from the other side". It is not clear to me what "other side" means in this context.

We have reworded the sentence:
In this work we approach the problem of seizure propagation differently: instead of attempting to invert pre-existing model of seizure propagation, we introduce a novel model designed with the inversion in mind.

R2.5 Results, line 92: I suggest that the authors add here what w_ij is (for readers that didn't read the Methods section).

We have extended the description in the text:
The seizure propagates through the brain network, represented as a weighted network W = {wij} ∈Rn×n, where wij stands for the connection strength from region j to region i, and n stands for the number of brain regions. We estimate this patient-specific structural connectivity from diffusion-weighted MRI images.

R2.6. Line 110: I suggest that the authors explain how the 90 sec were chosen and also explain why this arbitrary threshold should not make an impact on their analysis.

*To answer the multiple related comments concerning the temporal scale of the modeled seizures (R2.6, R2.15, R3.1b, R3.7a), a paragraph motivating the exclusion of the seizures below 30 seconds and the time limit of 90 seconds was now added (**Materials and Methods/Dynamical model of seizure propagation/Temporal scale of modeled seizures**).*
Temporal scale of the modeled seizures. Because the function f_q is positive, the model implies that every region will start to seize at some finite time. In reality, this is not the case, and to account for that in the statistical model we introduce a time limit of a seizure t_{lim}, and consider all regions with onset time larger than the limit to be non-seizing. Setting this constant however poses a challenge, as it is influenced by two contradictory considerations. First, we want tlim to be high enough to avoid losing information, since any region with detected onset occurring after t_{lim} is considered non-seizing, and the exact timing information is lost. At the same time we however want tlim to be also low: the model does not model seizure offset, and assumes that every recruited region stays seizing (and possibly pushing the connected regions to seizure state) until time t_{lim}. This assumption is broken for every region which terminates its seizure activity before t_{lim}, therefore low t_{lim} is desirable as well. No perfect solution exists to this conundrum, particularly considering that the constant is shared for all seizures in the data set in the present version of the model. We set t_{lim} = 90 s, so that the interval between the first detected onset time and the seizure limit ($t_{lim} - t_1$ = 60 s) approximates the median duration of all seizures in our data set (65.95 s). This choice expresses the time scale that we consider relevant for seizure propagation. Furthermore we excluded from our data set all seizures shorter than half of that (i.e. 30 s) to avoid

contaminating the data set with seizures where the assumption that every region seizes until t_{lim} is most glaringly broken.
In the future, this issue could be partially alleviated by seizure-specific models with varying tlim, we consider it in Discussion.

R2.7. Lines 121-122, the authors wrote: "we want to infer the parameters q of the excitation function, which we assume are shared among all seizures". I suggest that the authors further clarify that they mean “all” the seizures from all the patients (and not all the seizures of each individual separately). The authors may also add that this is assumed for the sake of simplicity (and tractability?).

The matter was clarified in the main text, and reference to Discussion where the issues is discussed was added:
*[...] we want to infer the parameters **q** of the excitation function, which we assume are shared among all seizures and for all patients, and the seizure-specific excitabilities. This assumption is made for the sake of simplicity; we discuss the limitations of it in Discussion.*

R2.8. Lines 149-150, the authors wrote: "we observe that the state of over 90% of the observed regions is unambiguous". It is then clarified in the caption of Fig. 3 how the ambiguity is dealt with. I suggest that important details such as this that are stated in the figures’ captions should be either copied or moved to the main text (more examples below).

The clarification was added to the main text:
[...] we observe that the state of over 90% of the observed regions is unambiguous (Fig. 3E). In the remaining cases, the seizing or non-seizing status is determined based on the majority of channels.

R2.9. In the caption of Fig. 3, the authors wrote: " (H) Detected onset times of all seizing regions, relative to the clinically marked seizure onset." – I suggest that the authors further clarify that this is a time difference. The same applies to (I).

The captions were reworded:
(H) Difference of the detected onset times of all seizing regions from the clinically marked seizure onset. (I) Difference of the detected onset times of the first seizing region of every seizure from the clinically marked seizure onset.

R2.10. I suggest that the authors explain briefly what q_aa, q_ab, q_ba, and q_bb are in Fig. 4 (or in the accompanying text) for the convenience of readers that have not read the Methods section.

A brief explanation was added to the figure caption:
*Figure 4: [...] (A) Posterior (full lines) and prior (black dashed lines) distributions of the hyperparameters for the models fitted with two data folds. Hyperparameters **q** = (qaa, qab, qba∗, qbb∗) parameterize the right-hand side function f**q** of the dynamical model of seizure propagation (1).*

R2.11. The number of effective samples is introduced in the paragraph in lines 165-170, but its significance and interpretation are not presented.

The description and interpretation of the number of effective samples was added to the text: Number of effective samples N_eff is the estimate of the number of independent samples with the same estimation power as the obtained autocorrelated samples. Here in some cases the R^ values lie slightly outside of the generally recommended range R^ < 1.1 indicating imperfect mixing of the MCMC chains; we consider it acceptable given the practical limitations of the required time for the computations and considering that the hyperparameter posterior distribution will be reduced to point estimate for the second step of fitting the individual seizures. For the same reason we also do not consider the low number of effective samples problematic.

R2.12. The threshold c_h=2 is introduced without explanations. I suggest that the authors explain its meaning, and how they chose this specific value. It may also be relevant to show how this threshold may impact on their results.

The motivation for choosing the value was added to ***Materials and Methods/Evaluation measures/Precision and recall****.*
The value of $c_h = 2$ was chosen based on the visual inspection of the results as a value that sufficiently identifies the highly excitable regions (see examples in Fig. 5 in the main text and Fig. 7 and Fig. 8 in S1 Text). The regions with $c > 2$ are capable of autonomously switching to seizure state, that is, without any input from other recruited regions. As a reference, the threshold $c_h = 2$ corresponds to 3.69 epileptogenic regions out of 162 if the excitabilities the prior of standard normal distribution.

R2.13. Concerning the results presented in Fig. 6, is the state prediction and onset prediction accuracies dependent on the location of the region being predicted? One could expect that regions on the boundary of the mapped regions by intracranial EEG may be more difficult to assess. If not, then this may add further support for the proposed method. I suggest that the authors address this possibility.

The analysis of the influence of region location was added to ***Fig. 6.*** *Specifically, the feature* ***Node strength (obs.)*** *represents the connection strength to and from the observed regions, thus quantifying if a region is strongly ("core" of the mapped regions) or weakly ("boundary" of the mapped regions) connected to other observed regions. The importance of this feature is low, indicating that no strong relation between the region location with respect to implantation exists.*

R2.14. I suggest to the authors to further interpret and explain the results presented in Fig. 7E in the text (around lines 252-255).

More detailed description and interpretation of the results was added to the main text. In this reply, the revised text is cited in full under the comment R1.3.

R2.15. Methods, lines 412-413, the authors wrote: "Only the seizures with duration longer than 30 seconds were included in the analysis." – I suggest that the authors add a justification as for why they neglected shorter seizures.

*To answer the multiple related comments concerning the temporal scale of the modeled seizures (R2.6, R2.15, R3.1b, R3.7a), a paragraph motivating the exclusion of the seizures below 30 seconds and the time limit of 90 seconds was now added (**Materials and***

Methods/Dynamical model of seizure propagation/Temporal scale of modeled seizures). *The paragraph is copied in full under R2.6.*

R2.16. At the beginning of section 4.4, the authors define low and high frequencies and distinguish between the two based on frequencies being lower or higher than 12.4Hz. Why 12.4Hz specifically?

The value 12.4 Hz represents the border between the alpha and beta bands. The motivation for this separation based on the use of theta-alpha and beta-gamma bands for quantifying the epileptogenicity of brain structures was added to the text:
First, the log- power in two frequency bands (LPlow(t) from 1 to 12.4 Hz encompassing the θ and α bands; LPhigh(t) from 12.4 to 100 Hz encompassing the β and γ bands) was calculated by computing the time-frequency representation using the multitaper method, summing over the given frequency band. This division follows the use of the two bands in quantifying the epileptogenicity of the brain structures (Bartolomei et al., 2008).

R2.17. From my understanding, the equation for the LP^norm should take the absolute value of the RHS (at the beginning of page 18).

We define the LP^norm(t) simply as LP(t) shifted by the preictal baseline. No absolute value is therefore needed.

R2.18. Line 488, the authors wrote: "…we did not assign the observation to any region" – I was surprised by this. Why not assign it to both regions? I suggest that the authors justify their choice.

We added the motivation to the text:
We opted for this cautious approach to minimize the risk of assigning the observation to the wrong source, guided by the rule that smaller amount of reliable data is preferable to larger amount of unreliable data.

R2.19. I suggest to the authors to further motivate and explain the parameterization function (lines 519-526).

The paragraph ***Materials and Methods/Dynamical model of seizure propagation/Parameterization of the excitation function*** f_q *was revised and extended, and a sketch to illustrate the parameterization was added.*

R2.20. I commend the authors for their interesting Supplementary Material. I suggest to the authors to add more mentions to it in the main text, particularly to the results presented there (pages 30 to 36).

A description of the results presented in ***Supplementary information*** *was added to* ***Results/Overview of the method***:
Due to the simplicity of the dynamical model, mathematical statements about the existence and uniqueness of the solution of the inverse problem can be made in case of no observation error (S1 Text/Note on parameter identifiability). In particular, we show that a solution of the inverse problem is guaranteed to exist for any combination of onset times of the seizing nodes. In fact, in the network with nns non-seizing nodes, nsz seizing nodes, and

nhid hidden nodes, there exist infinite amount of solutions, represented by (nns − nhid)-dimensional manifold in the parameter space.
Furthermore, the model can be robustly inverted even for large brain networks, as we demonstrate on extensive validation with synthetic data (S1 Text/Validation on synthetic data). We show that the hyperparameters ***q*** *of the model can be recovered in multiple scenarios, and we illustrate the limits of the method regarding the recovery of the region-specific excitabilities* ***c*** *for both observed and hidden regions. We also evaluate the capacity of the model to discover the epileptogenic zone which is not explored by the implantation, and demonstrate the role of the network connections in this effort (S1 Text/Discovery of epileptogenic zones in synthetic seizures).*

Reviewer #3: Sip et al. investigate if a simple computational model of spreading activity simulated on patient-specific structural (white matter) networks can explain observed patterns of seizure spread in different seizures across a cohort of epilepsy patients. Although the validation of the results may be negative, I still find this a very instructive study that I enjoyed reading, and that deserves to be published.

I have some general comments for the authors, followed by technical questions that I hope will help the authors strengthen their results and improve their paper.

General comments:

R3.1. Figures 3G & 4B: One striking observation in both figures is that a disproportionate amount of seizures have all regions seizing at some point.
R3.1a. I suspect these are secondary generalised seizures? These seizures should perhaps be excluded as the mechanism of the generalisation is most likely thalamo-cortical, which you do not explicitly model. I suspect this is also the reason for the mismatch in simulated vs observed data in Fig 4B 1st panel? If you do not have that information on the seizures, I think it is at least worth a discussion point.

*Indeed, some seizures were secondarily generalized (**Table 3 in Supplementary information** now shows that). For the reasons of computational costs we did not repeat the study with the seizures excluded, however, in the added analysis of the leave-one-out validation results (**Fig. 6C**) we checked whether the state and onset prediction accuracies differ for the secondarily generalized seizures. The results do not reveal such relation.*
*Nevertheless, this analysis does not eliminate the problem that the model was first trained on all seizures. This general issue is discussed in **Discussion/Shared seizure dynamics**.*

R3.1b. The arbitrary cut-off of 90 seconds (to determine if a channel is seizing) is rather strange. Why can you not use the actual seizure duration as the cutoff in the seizure-specific models?

*To answer the multiple related comments concerning the temporal scale of the modeled seizures (R2.6, R2.15, R3.1b, R3.7a), a paragraph motivating the exclusion of the seizures below 30 seconds and the time limit of 90 seconds was now added (**Materials and***

Methods/Dynamical model of seizure propagation/Temporal scale of modeled seizures). *The paragraph is copied in full under R2.6.*

In addition, we point out that in this work we do not use seizure-specific models (that is, models where hyperparameter vector ***q*** *is seizure-specific). We instead use only one model (defined by the hyperparameter vector* ***q****) for all seizures. We however now emphasize and discuss the question of seizure-specific models in* ***Discussion/Shared seizure dynamics****.*

R3.1c. Related to this, it may be worth showing in another figure/panel what Fig 4B would look like using seizure-specific models?

We agree that seizure-specific models are worthwhile research direction (and it is identified as such in ***Discussion/Shared seizure dynamics****), but outside of the scope of the work.*

R3.2. Figure 6 validation results. Overall it is unclear to me if these distributions represent the overall distribution across all validation iterations in each seizure in each patient, or some aggregate across iterations/seizures/patients? I have several related comments for this aspect:

R3.2a. I simply don't understand where n=4542 and n=1679 come from. Is this related to lines 214 & 215? If so, please clarify in the text.

First, we note that the value n=4542 was introduced by a bug in the analysis code. That issue was fixed. Second, we have clarified the nature of the data points in the figure label, where we now state:
In the upper panel, each data point ($n = 2863$) corresponds to one observed region in one seizure ($n_{seizures} = 137$) of one patient ($n_{patients} = 44$) for which LOO analysis was successfully performed. In the lower panel, only the seizing regions are considered ($n = 1679$).

R3.2b. Would patients with many seizures, or many electrodes not bias the distribution shown?
*Yes. The results show the accuracy distributions across the whole dataset (which is not homogeneous concerning the number of seizures per patient), and do not purport to show any "patient-averaged" predictive distribution. However, the new feature importance analysis of the results (****Fig. 6C****) does not indicate that strong patient-specific effects that would bias the results are present. We comment on this in the main text:*
It is worth noting that the distributions of prediction accuracies are constructed from all observed regions in all available seizures, and some patients have more recorded seizures than others. The distribution thus do not show any "patient-averaged" prediction accuracy, rather the performance on the whole data set. Nevertheless, the following results indicate no patient-specific effects that would bias the distribution are weak.

R3.2c. It is well-known that patient-specific effects exist, so an analysis on the patient level would greatly help understanding the data. For example, are the validation accuracies particularly low for patients with worse surgical outcomes? Is it related to any other patient level feature you list in table S3? Do other aspects like implantation design influence the validation accuracies?

*The new feature importance analysis of the leave-one-out validation results in **Fig. 6C** now includes the patient specific effects, and the results are discussed in the main text. In particular, we analyzed patient-level features Engel score, epilepsy type, MRI lesion findings which do not have strong effect on the accuracies. As for the implantation design, we observe decreased performance for nodes with low node strength.*

R3.2d. Seizure-specific properties have also been highlighted recently, and perhaps it is worth investigating if the same patient can have vastly different validation accuracies for their seizures? Could this serve as a tool to select one specific seizure per patient for the subsequent analysis on surgical outcome (Fig 7)? Clinically, one would not necessarily expect all seizures in a patient to be informative for the surgical strategy.

*The new analysis of the leave-one-out validation results in **Fig. 6C** now includes also the seizure specific effects. From these, the method identified only the fraction of seizing nodes among those observed as a relevant factor for predicting the accuracies. This is discussed in the revised associated main text.*

Regarding the selection of a specific seizure for a surgery outcome analysis: Our goal here is to test the method on the whole dataset; the opposite could be easily interpreted as cherry-picking suitable data to get desired results (even if well motivated by the LOO validation results). We agree that clinically not all seizure might be informative, but the method should be able to account for that: when the data are not informative, the inferred posterior distribution should indicate this uncertainty, and vice-versa, thus automatically identifying the informative seizures.

R3.2e. Leave-one-out validation may be a bit too "weak" in this analysis, as very often, a single channel can simply be predicted by its neighbouring channels. This is also possibly why the model inference does not outperform the weighted null-model. You may get stronger results when you leave out an entire electrode shank, which is also simulating a more realistic clinical scenario.

*The leave-one-out validation is not done on the sensor level (channels/electrodes), rather on the source level (brain regions). Specifically, we leave out a single brain region (to which more than one channels are often projected, see **Fig. 3D**). This approach allows the focus of the LOO validation on the role of the network in the prediction; LOO validation on the sensor level could not achieve that. We have commented on this in the main text:*
We note that the LOO analysis is done on the region (and not the channel) level. Given that multiple contacts of a single electrode (or several electrodes) are often assigned to a single region, this approach is loosely comparable to leaving out multiple channels at once, with the benefit of allowing us to focus on the role of the structural network (and not the source-to-sensor projection) in the prediction.

R3.2f. I think the results are also slightly overstated/overgeneralised in lines 224 – 227. Perhaps being more specific would help. E.g. "The results demonstrate that we can predict if a channel seizes at any point in the seizure with a median accuracy of 80.5%." Etc. Of course, if these results are driven by particular patients/seizures, please revise accordingly. I feel similarly about other parts of the writing (e.g. abstract).

The statement was revised to the following:

The results demonstrate that the seizing/non-seizing state of a region can be predicted with a median state prediction accuracy 80.5%, and the onset time can be predicted with median onset prediction accuracy 29.9% (Fig. 6A). On group level, these values are however not better than those of both the unweighted and the weighted estimates (Fig. 6B).

The statement in the abstract was revised to the following:
Using a leave-one-out cross-validation scheme on a cohort of 45 patients we demonstrate that the method can improve the predictions of the states of the unobserved regions compared to an empirical estimate that does not use the structural information, yet it is on the same level as the estimate that takes the structure into account.

R3.3. In Figure 7D and all the results relating to surgical outcome, have you considered the effect of the size of the resection, or the total number of nodes removed? There are also some indications in the literature that size of resection may be related to surgical outcome, have you tested this? Other authors create a null-model for each patient (often performed as virtual "random resections" of the same size as the actual resection) to tackle this problem. It is hard to appreciate/interpret the results in this part of the paper otherwise.

*Here we investigate whether we can predict the surgery outcome of the actual surgery by performing the exact same surgery in the trained in-silico model. If the actual surgery outcome is influenced by the size of the resection, the in-silico surgery outcome should reflect this, but that is already tested by our approach. To address this point, we have now added the analysis of the relation of actual/in-silico surgery outcome and the resection size (**Fig. 12 in Supplementary information**), and commented on the results in the main text:*
Neither the real nor the virtual surgery outcome shows a strong dependence on the number of resected regions in the examined patient data set (Fig. 12 in S1 Text).

*Furthermore, we have extended the description of the analysis presented in **Fig.** 7 in the main text to clarify its goals:*
In addition to the predictions of the onset times in hidden regions, the product of the inference is also a spatial map of the excitability parameter c for every seizure. We evaluated whether this excitability is useful for localizing the epileptogenic zone. No ground truth to directly validate against exists, therefore we employed the following methodology: we restricted ourselves only to the patients that were operated and for which the post-operative MRI was available. We extracted which brain regions were resected, and using the inferred excitabilities for a specific patient and a specific seizure we performed an in silico resection, that is, we removed the resected regions from the network model and we performed the forward simulations with the excitabilities inferred from the observed seizures (Fig. 7A-C). If the fitted model represents the reality well, one can expect that the successful surgeries that stopped the seizure occurrences in the patient will stop the seizures also in the computational model, and that the failed surgeries that did not reduce the seizure occurrences will fail in the computational model too. In other words, real-world outcome classified by the Engel score should correlate with in silico outcome.

Technical questions:

R3.4. When were the surgical outcomes assessed?

*The information was added to **Methods/Patients and data acquisition**.*
Postoperative seizure outcome was assessed at the latest available follow-up according to the Engel classification (Engel, 1993) i.e. at one year or later following surgery.

R3.5. Two different imaging protocols were used. A quick report that the main results did not differ by protocol should be made.

*We have added the report to **Results/Structural connectomes**:*
The imaging protocols differed between the patients, most notably in the number of gradient directions in diffusion-weighted imaging (64 or 200). To assure that this did not affect the study, we compared the two groups in terms of several network properties of the generated structural connectomes. The connectomes did not significantly differ between the two groups ($n1 = 27$, $n2 = 23$) in terms of mean node strength (Mann-Whitney U test, $U = 264$, $p = 0.185$), mean clustering coefficient ($U = 245$, $p = 0.103$), or edge density (binarization threshold 0.001, $U = 289$, $p = 0.341$). Difference was observed in terms of network modularity ($U = 175$, $p = 0.004$), but with modest effect size (mean modularity in the two groups 0.450 and 0.427).

R3.6. I did not understand the reasoning behind the assignment of the channels to brain regions. "If the two nearest regions were in a similar distance from the midpoint (i.e. satisfying $d2/(d1 + 0.5mm) < 2$, where d1 and d2 are the distances of the nearest and second nearest region respectively), we did not assign the observation to any region, otherwise we assigned it to the closest region". In an example: you wouldn't assign a channel with d2=2 and d1=1 ($2/(1+0.5)=4/3<2$) to any region, but you would assign a channel with d2=20, d1=9 ($20/(9+0.5)\sim=2.1>2$) to the nearest region that is in this case 9 mm away? I don't think a simple ratio is the way forward here, was this a typo?

It is not a typo, and if this scenario would occur, it is exactly what would have been done: the goal is to simply find the closest region and avoid assigning a channel to any region in ambiguous situations. We added the motivation to the main text:

We opted for this cautious approach to minimize the risk of assigning the observation to the wrong source, guided by the rule that smaller amount of reliable data is preferable to larger amount of unreliable data.

We agree that this approach is quite simplistic and more advanced sensor-to-source projection approach could be beneficial, however, at this stage of the exploration of this novel method we preferred an approach which is simple and easily interpretable.

R3.7. "Only the seizures with duration longer than 30 seconds were included in the analysis. For some patients only these short seizures were recorded; their structural data were used in the study nevertheless."
R3.7a. The reasoning behind excluding seizures of less than 30 s is unclear. Depending on the pathology and patient, 25 s long seizures are perfectly "valid" and informative. Especially If they are the only type of seizure in a patient.

*To answer the multiple related comments concerning the temporal scale of the modeled seizures (R2.6, R2.15, R3.1b, R3.7a), a paragraph motivating the exclusion of the seizures below 30 seconds and the time limit of 90 seconds was now added (**Materials and***

Methods/Dynamical model of seizure propagation/Temporal scale of modeled seizures). The paragraph is copied in full under R2.6.

R3.7b. Can you please also clarify if you generally considered all seizures in a patient? If not, what were the selection criteria?
R3.7c. Were subclinical seizures included?

The two questions are now addressed in the text:
Only the seizures with duration longer than 30 seconds were included in the analysis, and subclinical seizures were not considered. Apart from that, no other criteria for exclusion were applied.

R3.8. If I understand you correctly, you use the structural connectivity only for some patients. This is only for the analysis of the multi-seizure model? If so, please clarify here.

The question is now addressed in the text:
Some patients in the cohort did not have any seizures satisfying the duration criterion. Their structural connectomes, described in the following section, were constructed nevertheless, and they were used for generating and analyzing synthetic seizures (S1 Text/Validation on synthetic data).

R3.9. In the first part of results, please clarify what q is exactly, as you then report on qaa, qab etc. without further explanation. Simply changing the order of Methods and Results may also be helpful. It may also help the reader if in the schematic Fig 1C you clarify the role of q and c.

*We clarified the role of hyperparameters **q** in the caption of **Fig. 4**, where these are discussed:*
*Figure 4: [...] (A) Posterior (full lines) and prior (black dashed lines) distributions of the hyperparameters for the models fitted with two data folds. Hyperparameters **q** = (qaa, qab, qba∗, qbb∗) parameterize the right-hand side function f**q** of the dynamical model of seizure propagation (1).*

R3.10. Suggest to clarify if the cortical parcellation used contains ROIs of roughly equal size and its implications.

*We added a reference (in **Materials and Methods/Structural model of the brain**) to the recently accepted paper (Wang et al., 2020) describing in detail the custom atlas employed here. In particular, the paper analyzes the size of the ROIs of the custom parcellation.*
Due to the splitting of the largest cortical regions from the Destrieux atlas, the resulting parcellation contains cortical regions with sizes on the same order of magnitude (Wang et al., 2020). While the method can be applied to parcels of uneven size, vast differences in sizes could lead at the same time to high computational costs (spent on the small regions) and low precision (limited by the large regions).

R3.11. "At most two seizures per single patient were used for the fitting [of the multi-seizure model I presume] to avoid biasing the model towards the patients with more recorded seizures.": How did you select the two seizures? For patients with only one seizure: is that not biasing against them?

We now state the method of selection in the text:
If more than two seizures were available for one subject, the two seizures were selected randomly.

The reason for using at most two seizures is to avoid domination of the dataset by a set of similar seizures from a single patient. Imagine we have 10 patients with a single seizure, and one with 10 very similar seizures. With no limit we would train the model with 10 similar seizures out of 20 seizures, which might bias the trained model to predict well those 10 seizures. If we put the limit on at most two seizures, we would have at most two similar seizures out of 12, and the model, presumably, should generalize better. We summarize this argument in the reworded text:
To facilitate better generalization of the trained model to unseen seizures and to avoid the possibility of the training set being composed of large amount of similar seizures, we included at most two seizures from a single subject.

R3.12. "In our model we explain this variability by the seizure-specific excitabilities, and, to a lesser degree, by patient-specific structural connectomes." Can you clarify when you assessed the role of the patient-specific connectome? This is an interesting point!

While the patient-specific connectomes were used in the study, their role was not explicitly assessed. We reworded the sentence to the following:
In our model we allow this variability to be explained by the seizure-specific excitabilities in conjunction with the patient-specific structural connectomes.

R3.13. Related to the previous point: Line 437-439: dividing wij (fibre count) by target region volume may be problematic. Target region volume covaries with total brain volume. The target region may also show patient-specific patterns of atrophy. Therefore, these networks may not be comparable across subjects. Please also clarify if log fibre count was used.

The log fibre count was not used, as we now explicitly state in the text:
Each element w^ij of the generated connectome matrix W^ represents the number of fibers from region j to region i; note that we did not use the log-count of the fibers.

The structural connectome matrices were normalized in order to make them comparable, as we state in the text:
Finally, the connectome is normalized so that the maximal sum of ingoing projections is equal to one.

R3.14. Section 4.9: please clarify N.

We have now clarified N in the text:
[...], N is the number of posterior samples, [...]
