# Supplementary material for: Data-driven method to infer the seizure propagation patterns in an epileptic brain from intracranial electroencephalography
Source: PLoS Comput Biol. 2021 Feb 17;17(2):e1008689. doi: 10.1371/journal.pcbi.1008689 (PMC7920393; doi:10.1371/journal.pcbi.1008689)
Supplement: Attachment — Submitted filename: ResponseLetter_rev2.pdf [file pcbi.1008689.s003.pdf]

Manuscript PCOMPBIOL-D-20-01581R1
Response to reviewers

Dear Dr Marinazzo,

We submit the second revision of our manuscript titled “Data-driven method to infer the seizure propagation patterns in an epileptic brain from intracranial electroencephalography”. Once again, we would like to thank the reviewers for careful reading, constructive criticism, and useful suggestions.

Attached is the revised manuscript, and a version with the changes highlighted.

Apart from the changes arising from reviewers’ comments described below, we have corrected the terminology in Box 1 and Box 2 inconsistent with the text (“Parameters” → “Constants”; added item “Parameters”).

As before, we employ the following conventions in the replies:

- Original reviewers’ comments are in black.
- *Our replies are in green italics.*
- ***References to sections, figures, or tables in revised manuscript are in bold green italics.***
- *Full or partial elements of the revised text are in red italics.*

Reviewer #1: The authors have addressed my comments. Thank you.

We thank for the thoughtful comments and questions.

Reviewer #2: The authors have answered all my queries adequately.

We thank for the useful queries.

I would like to just point out a few minor corrections:

1) Section 2.5, 1st paragraph: "epileptognic" -> epileptogenic

The typo was fixed.

2) Section 2.6, the authors wrote: "Nevertheless, the following results indicate no patient-specific effects that would bias the distribution are weak." – I find the sentence hard to read.

The sentence was corrected to:
Nevertheless, the following results indicate that the patient-specific effects that might bias the distribution are weak.

3) Section 2.7, penultimate paragraph: "worst result" – I suggest adding what "worst result" means.

The sentence was rewritten as:
[...] and when taking both the average or minimal value of the relative reduction across seizures for patient when multiple seizures were available (Fig. 11 in S1 Text).

4) Section 3.3: the authors wrote: "Given that the method is trying the fill in incomplete data," – I assume the authors meant to write "to fill" instead of "the fill".

Indeed. The typo was fixed.

Reviewer #3: I thank the authors for addressing some of my comments, but a few points remain open.
I don't typically like knit picking if the details don't have a great impact on the overall conclusions. However, in this case, it's hard to estimate the impact of the methods on the overall results. In general, given that this paper will likely influence future generations of researchers, I wouldn't like to see methods being reused in future that may be suboptimal.
Here, I highlight one main open issue, and suggest simple ways to address two other points. I would still like to express my enthusiasm for the overall approach though and hope the authors find the following useful.

We thanks for the past and current comments and we answer the current ones below.

Main issue: Previously I highlighted the issue of reporting aggregate statistics across iterations/seizures/patients, with e.g. different numbers of seizures per patient. In my opinion, the statistically appropriate way to report the results is using hierarchical approaches that can account for the different nested levels of the data. Please report all statistics in this manner where applicable. Data visualisations should also acknowledge the nested nature of the data, where applicable. I realise this may not change your conclusions dramatically, but given the rising interest in subject-specific modelling, your paper will be setting an example and precedence in terms of the methods and techniques.

*We have added the analysis of the leave-one-out validation results using a multi-level model in order to compare the differences between subjects and seizures (**SI Text/Subject-level analysis of the prediction accuracy** and **Figures 13 and 14 in SI Text**). The analysis shows the existence of the inter-subject differences, however, detailed analysis of these differences and their causes remains out of scope. In the main text we have referenced the added analysis with the following:*

Finally, we have analyzed the results on a subject and seizure level using a multi-level hierarchical model that accounts for the discovered dependency on the node strength and fraction of seizing regions in a seizure (S1 Text/Subject-level analysis of the prediction accuracy). Results are presented on Fig. 13 and Fig. 14 in S1 Text.

The analysis shows that individual variation in accuracies remains even after the accounting for the node strength and fraction of seizing regions, both at the subject and seizure level. Detailed analysis of the variations is out of scope of this work, nevertheless, these results can guide the future investigation of the model strengths and weaknesses on the subject and seizure level.

On the topic of statistical reporting, please also review the paper for accurate statistical reporting throughout. One example is "Difference was observed in terms of network modularity (U = 175, p = 0.004), but with modest effect size (mean modularity in the two groups 0.450 and 0.427)." Mean is probably not a useful measure of effect size here?

We now report more descriptive statistics and we replaced mean with median more consistent with Mann-Whitney test:

Difference was observed in terms of network modularity (U = 175, p = 0.004), but with modest effect size (min/median/max modularity in the two groups 0.394/0.451/0.497 and 0.381/0.424/0.498).

If we misinterpreted the comment and the concern was instead with the use of unstandardized effect size measure as opposed to a standardized measure such as r value, we argue that the absolute values of modularity are more interpretable to readers, especially those interested in this particular section, familiar with connectivity measures and their interpretation.

R3.6: "If the two nearest regions were in a similar distance from the midpoint (i.e. satisfying d2/(d1 + 0.5mm) < 2, where d1 and d2 are the distances of the nearest and second nearest region respectively), we did not assign the observation to any region, otherwise we assigned it to the closest region. We opted for this cautious approach to minimize the risk of assigning the observation to the wrong source, guided by the rule that smaller amount of reliable data is preferable to larger amount of unreliable data."

I previously asked you about a hypothetical scenario: "you wouldn't assign a channel with d2=2 and d1=1 (2/(1+0.5)=4/3<2) to any region, but you would assign a channel with d2=20, d1=9 (20/(9+0.5)~=2.1>2) to the nearest region that is in this case 9 mm away?"

I still think you at least require a minimum distance before you apply your ratio criterion. Electrode contacts buried deep in white matter, far away from cortex are very unlikely to record a pure signal from a single region, regardless of what their ratio of d1 and d2 is. I think with your ratio method you are actually adding more unreliable data (in my example by including signals recorded far away from cortex). As a way forward, I'd suggest reporting the proportion of contacts that are e.g. more than 3mm away, and add a note to the methods and code that this should be re-visited in a future study.

The concern is now clearer to us. We have removed the previously added sentence, and added the following:

Note that we did not set any upper limit on the distance, and thus, in theory, contacts arbitrarily deep in the white matter with little relevant signal could be assigned to a brain region. In our study 99.3% of the assigned contacts were less than 3 mm away from the gray matter tissue and the furthest was 4.47 mm away, so we do not consider the issue particularly problematic here, however more cautious approach should be employed in future studies.

R3.11:"To facilitate better generalization of the trained model to unseen seizures and to avoid the possibility of the training set being composed of large amount of similar seizures, we included at most two seizures from a single subject." Maybe this is a misunderstanding, but by your logic, including one seizure per subject would be best, no?
Perhaps the easiest way forward is to simply state something like: "This resulted in the inclusion of xx patients with 2 seizures and yy patients with 1 seizure."

We have clarified the motivation and stated the number of included patients:

We included at most two seizures from a single patient in the learning data set. We opted for two seizures as an imperfect compromise between the need for good generalization of the trained model (to which including large amount of possibly similar seizures from a single patient would be detrimental) and the wish to include as much data as possible. In the future, the optimal way to deal with uneven number of seizures would be to build a hierarchical model with subject and seizure levels that can account for that. If more than two seizures were available for one subject, the two seizures were selected randomly. This resulted in the inclusion of 21/18 patients with two seizures and 1/4 patients with one seizure in the first/second fold.
